# Mice produced by mitotic reprogramming of sperm injected into haploid parthenogenotes

**DOI:** 10.1038/ncomms12676

**Published:** 2016-09-13

**Authors:** Toru Suzuki, Maki Asami, Martin Hoffmann, Xin Lu, Miodrag Gužvić, Christoph A. Klein, Anthony C. F. Perry

**Affiliations:** 1 Laboratory of Mammalian Molecular Embryology, Department of Biology and Biochemistry, University of Bath, Bath BA2 7AY, UK; 2 Project Group Personalized Tumor Therapy, Fraunhofer Institute for Toxicology and Experimental Medicine ITEM, 93053 Regensburg, Germany; 3 Experimental Medicine and Therapy Research, University of Regensburg, 93053 Regensburg, Germany

## Abstract

Sperm are highly differentiated and the activities that reprogram them for embryonic development during fertilization have historically been considered unique to the oocyte. We here challenge this view and demonstrate that mouse embryos in the mitotic cell cycle can also directly reprogram sperm for full-term development. Developmentally incompetent haploid embryos (parthenogenotes) injected with sperm developed to produce healthy offspring at up to 24% of control rates, depending when in the embryonic cell cycle injection took place. This implies that most of the first embryonic cell cycle can be bypassed in sperm genome reprogramming for full development. Remodelling of histones and genomic 5′-methylcytosine and 5′-hydroxymethylcytosine following embryo injection were distinct from remodelling in fertilization and the resulting 2-cell embryos consistently possessed abnormal transcriptomes. These studies demonstrate plasticity in the reprogramming of terminally differentiated sperm nuclei and suggest that different epigenetic pathways or kinetics can establish totipotency.

## Abstract (web summary)

It is unclear what regulates gamete reprogramming competence. Here, the authors inject sperm into parthenogenetic embryos, generating viable offspring and show that mouse embryos in the mitotic cell cycle can reprogram sperm for full term development.

## Graphical abstract

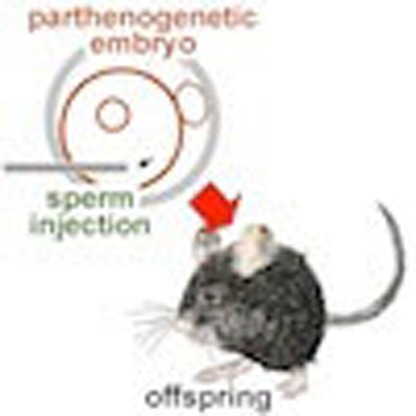

Fertilization integrates multiple cell-autonomous processes to transform a sperm and an oocyte (egg) into a totipotent embryo. In mammals, the fertilizing sperm triggers embryogenesis by inducing a calcium-dependent phospho-relay to remove the unique cytostatic factor, Emi2, resulting in metaphase II (mII) exit and completion of meiosis^1^,^2^. Sperm membranes, mitochondria and perinuclear matrix components are dismantled and the unique non-histone major sperm nucleoprotein, protamine, is replaced by maternal histones^3^,^4^,^5^. Both parental chromatin sets undergo signature patterns of global modification^2^,^4^. Paternal and, to some extent, maternal genomes undergo active 5′-methylcytosine (5mC) depletion^4^,^6^,^7^,^8^ accompanied by Tet3-catalysed production of 5′-hydroxymethylcytosine (5hmC). In the mouse, meiotic completion is followed by the formation of visible pronuclei (membranated compartments that uniquely separate each parental genome) ∼4.5 h after sperm entry; DNA synthesis and transcription respectively initiate after ∼8 and ∼10 h (Supplementary Fig. 1a,b)^2^,^5^.

Reprogramming during fertilization represents an extreme cellular potency change, from the assured death of sperm and oocyte to the emergence of the embryonic totipotent state able to engender an entire organism. Sperm are terminally differentiated, post-meiotic germ cells that are exhaustively remodelled during fertilization^4^,^5^ and nothing suggests that sperm are capable of being reprogrammed to totipotency by any other cell than oocytes, which are specialized for the role^9^. Sperm injected well after mII exit have so far failed to support development^10^,^11^,^12^,^13^,^14^. Although embryonic stem (ES) cell nuclei participate in development when injected into pro-metaphase 1-cell^15^ or interphase 2-cell^16^ embryos, they are pluripotent, contain diploid nucleosomal chromatin and are efficient nuclear transfer donors relative to somatic cells^17^. Since ES cells transit through a quasi-2-cell embryonic state during culture^18^, early embryonic reprogramming of their nuclei may be minimal. By contrast, sperm possess structurally unique toroidal chromatin whose principal nucleoprotein is protamine^3^. We therefore investigated whether embryos also possess the ability to reprogram differentiated cell nuclei exemplified by the inert, protaminic chromatin of sperm, to instate totipotency.

We now report that haploid parthenogenetic embryos injected with sperm undergo distinctive chromatin remodelling, including altered protamine-histone exchange kinetics, different 5hmC and histone modification dynamics, parental chromatin remodelling asynchrony and abnormal gene expression compared to normal fertilization. Nevertheless, this distinctive remodelling can result in full-term development. Our work suggests that sperm reprogramming sufficient to support full and healthy development can occur not only when mII oocytes are fertilized, but in mitotic embryos well after meiotic exit, via anomalous epigenetic pathways or kinetics.

## Results

### Development *in vitro* of parthenogenotes injected with sperm

**Figure 1:**
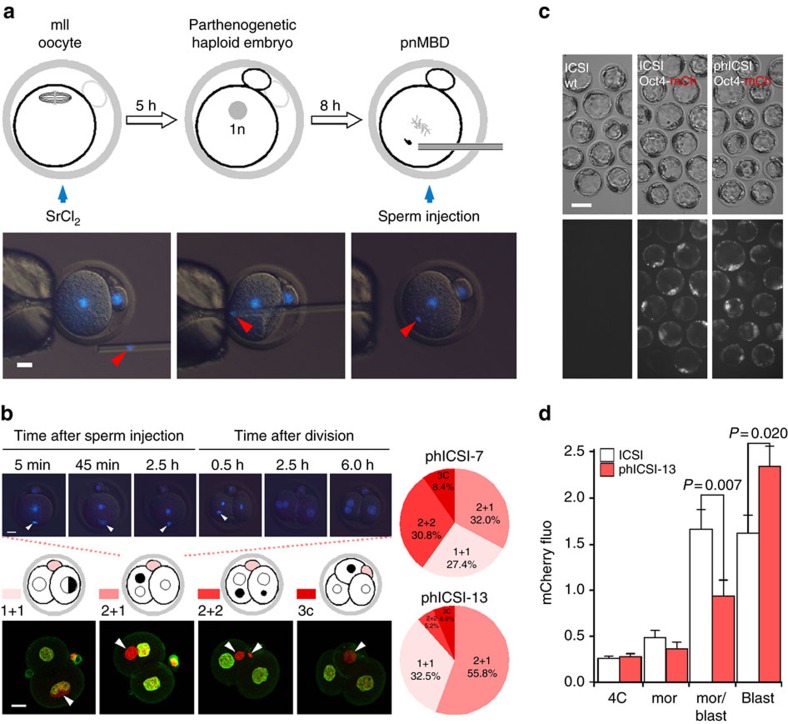
Development *in vitro* following phICSI-13. (**a**) Schematic (upper) and merged Hoffman and Hoechst 33342 (DNA) fluorescence images showing phICSI-13. Red arrowheads indicate sperm heads. (**b**) Merged Hoffman and Hoechst 33342 (DNA) images (upper) at the times indicated after sperm injection in phICSI-13. Schematic (centre: paternal genome, black; second polar body, pink) and fluorescence images (BrdU, maternal genome; PI, both genomes) show different classes of nuclear configuration 14 h after the first mitotic division (1C→2C) in phICSI-13. White arrowheads, paternal chromatin. Scale bars, 20 μm. Pie charts show proportions of each nuclear class in phICSI-7 (*n*=117) and phICSI-13 (*n*=77). (**c**) Hoffman (upper) and fluorescence micrographs of embryos generated by injecting mII oocytes (ICSI-1bla) or parthenogenotes (phICSI-13-1bla) with sperm carrying a transgene encoding mCherry expression driven by the *Oct4* promoter (*pOct4-mCherry*). Scale bar, 50 μm. (**d**) Histogram showing relative intensities of mCherry expression (±s.e.m.) of **c** in ICSI-1bla (open) and phICSI-13-1bla (red) embryos (*n*=25 [2C, blast] or 27 [4C, mor, mor/blast]). Experiments were on 2 days. Embryo stages are 4-cell (4C), morula (mor), morula-blastocyst (mor/blast) and blastocyst (blast). Differences are shown where *P*<0.05 (two-tailed, unpaired *t*-test).

We assessed the ability of parthenogenotes to reprogram sperm at different stages of the first cell cycle. We selected haploid parthenogenotes as our starting point so that the introduction of a sperm would potentially restore diploidy and rescue development. Parthenogenotes also allow us to eliminate potential contributions of paternal contributions earlier in fertilization and have previously been used to probe mitotic reprogramming of sperm^10^,^11^,^12^,^13^,^14^. Parthenogenetic haploid (ph) embryos generated by exposing mII oocytes to strontium chloride (SrCl_2_) were examined 7, 10 or 13 h after treatment started (ph-7, -10 and -13) (Fig. 1a; Supplementary Fig. 1a–c). SrCl_2_ induced rapid Geminin degradation typical of that seen in somatic cell G1 (Supplementary Fig. 1d) but differing from pluripotent cells in which Geminin escapes degradation^19^. Geminin and Cdt1 accumulated in the pronuclei of ph embryos, dispersing cytoplasmically after pronucleus (pn) membrane breakdown (pnMBD; Supplementary Fig. 1e). Following sperm injection (ICSI) of ph-7 embryos (phICSI-7), paternal chromatin remained compact with little morphological change for ≥5 h until the onset of maternal pnMBD (12–13 h after the start of SrCl_2_ treatment; Supplementary Fig. 2). By contrast, following ICSI of ph-13 parthenogenotes (phICSI-13) soon after pnMBD, sperm began decondensing within 1 h (Fig. 1b; Supplementary Fig. 2e), although spindle assembly on paternal chromatin was incomplete prior to the first mitotic cell division (Supplementary Fig. 2g, asterisk). The timing of cell division in phICSI-13 was similar to that of ph-13 controls (Supplementary Fig. 1c).

Cell division produced 2-cell embryos with four classes of nuclear configuration whose proportions differed between phICSI-7 and -13 (Fig. 1b); one class comprised 2-cell embryos with one biparental and one uniparental (maternal) blastomere (2+1). Altogether, 34.1–37.2% of phICSI embryos (*n*≥104) developed to the blastocyst stage, compared with 72.9% (*n*=85) for mock-injected haploid controls and 94% (*n*=84) in ICSI (Supplementary Table 1 and 'ph' in Supplementary Fig. 3a). Developmental failure in phICSI-13 largely occurred prior to the morula stage (29.5% of embryos) and during blastocyst formation (33.3%), but most (>55%) phICSI-7 and -10 embryos arrested during blastocyst formation (Supplementary Table 1). Sperm-derived *pCAG-mtdTomato*, *Nanog-GFP* and *pOct4-mCherry* transgenes were expressed in embryonic day 5 (E5.0) phICSI-13 blastocysts (Fig. 1c,d; Supplementary Fig. 4a–e), suggesting that reprogramming had enabled paternal genome activity. Ratios of Oct4- and Cdx2-expressing cells were normal in phICSI-13 blastocysts (Supplementary Figs 3b and 4f).

### Full development of phICSI embryos

**Figure 2:**
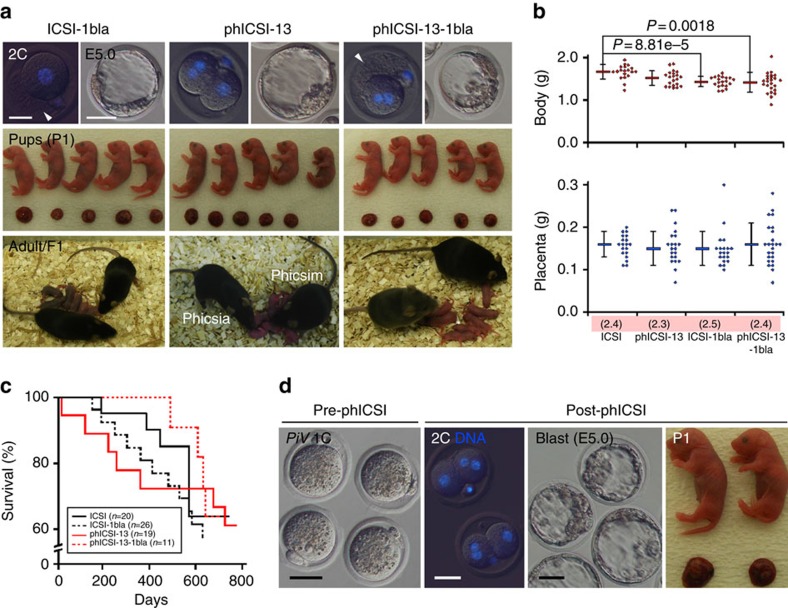
Full-term development of phICSI-13 offspring. (**a**) Fluorescence images (top) showing DNA stained with Hoechst 33342 in 2-cell stage embryos (2C DNA), Hoffman images of blastocysts (blast) at embryonic day 5.0 (E5.0); pups and their associated placentae (post-natal day 1, P1) and adults with F1 offspring (adult/F1). In ICSI-1bla and phICSI-13-1bla, a single blastomere was destroyed (arrowheads) at the 2-cell stage. Scale bars, 30 μm. (**b**) Plots of body (step value, 0.01 g) and placenta (step value, 0.01 g) mass in ICSI- and phICSI-13-derived offspring (1- and 2-bla) at term (±s.d.). Average litter sizes are given in parentheses. For ICSI, *n*=19; ICSI-1bla, *n*=20; phICSI-13, *n*=21; phICSI-13-1bla, *n*=24. Experiments were on ≥4 days per group. Differences are shown for *P*<0.05 (1-way ANOVA followed by Tukey–Kramer test). (**c**) Kaplan–Meyer survival plots of ICSI- and phICSI-13-derived offspring (1- and 2-bla). For ICSI, *n*=20; ICSI-1bla, *n*=26; phICSI-13, *n*=19; phICSI-13-1bla, *n*=11. (**d**) Parthenogenotes ectopically expressing transgenic Plcz (*PiV*) were injected with B6D2F1 sperm (1C), showing DNA staining (blue) at the 2-cell stage (2C DNA), blastocyst (blast) development after 5 days (E5.0) and two male offspring and their associated placentae at term (P1). Scale bars, 50 μm.

When 2-cell phICSI embryos were transferred to surrogate mothers, offspring of both sexes were born with overall efficiencies (that is, without eliminating aneuploid nuclear classes; Fig. 1b) of 1.0% for phICSI-7 (*n*=465 embryos transferred), 1.8% for phICSI-10 (*n*=384) and 8.1% for phICSI-13 (*n*=259; Fig. 2a, Supplementary Table 1; Supplementary Fig. 5a,b). Birth and placental weights in phICSI were in the control range, without evidence of fetal overgrowth, lifespan attenuation or other cloning-associated anomalies (Fig. 2b,c; Supplementary Fig. 5c); the first phICSI-13 survivors, a female (Phicsia) and male (Phicsim) were healthy and fertile (Fig. 2a), dying of natural causes after 1.5 and 2.5 years respectively. Sperm from the inbred strain, CBA/Ca, supported phICSI development (Supplementary Fig. 5d,e) and phICSI with spontaneously arising parthenogenotes (after pnMBD and approximately equivalent to ph-13) from *Plcz-ires-Venus* (*PiV*) transgenic females^20^ produced two (1.2%, *n*=154) healthy phICSI-*PiV* transgenic offspring (Fig. 2d; Supplementary Fig. 5f–h; Supplementary Table 1), demonstrating sperm reprogramming after autonomous parthenogenesis. These experiments show that mitotic embryos can support full-term development in phICSI and suggest that development was more efficient the closer sperm injection was to mitotic M-phase^21^.

To track lineage contributions of uniparental and biparental blastomeres in phICSI, we injected sperm carrying a ubiquitously expressed *Cre* transgene into *flox*ed *membrane tdTomato* (*mtdT*) parthenogenotes (Supplementary Fig. 6)^22^. In the resulting phICSI-mTmG^Cre^ embryos, exclusively maternal lineages fluoresce red, but paternally encoded Cre excises *tmdT* in biparental cells so that membrane-GFP (mGFP) is expressed instead (Supplementary Fig. 6a,b). Most cells in phICSI-mTmG^Cre^ fetuses were mGFP-positive but with detectable *mtdT* DNA at E11.5. Offspring expressed mGFP with no detectable *mtdT* DNA (Supplementary Fig. 6c–g), indicating that only biparental cells persisted at term.

To address whether uniparental lineages were necessary to complement defects early in development, we ablated the uniparental 2-cell blastomere of phICSI-13 (2+1) embryos (phICSI-13-1bla; Fig. 2a). 10.4% of phICSI-13-1bla embryos (24.0% of controls; 10.4 versus 43.4%) developed to term, with a similar rate for CBA/Ca sperm (Supplementary Table 1; Supplementary Fig. 5d,e). Thus, phICSI engendered totipotency, producing biparental blastomeres that supported full development at readily detectable rates.

### Full development by spermatid injection into parthenogenotes

**Figure 3:**
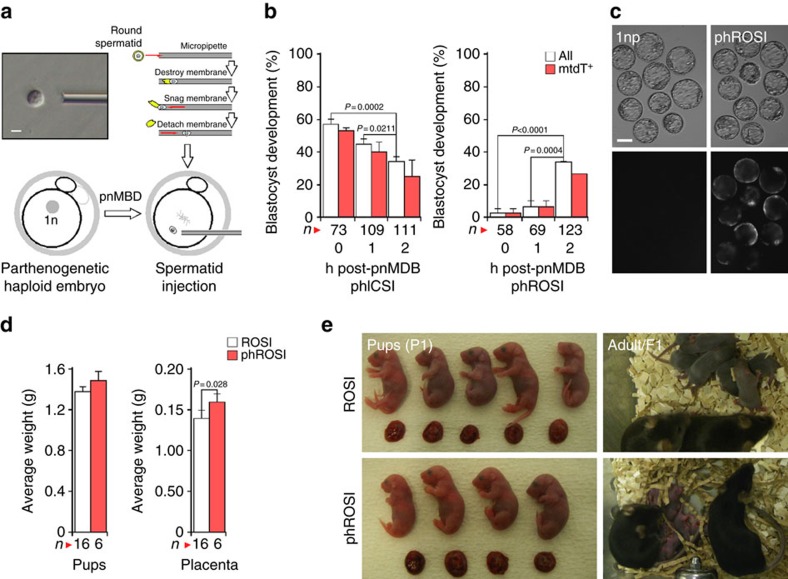
The phICSI protocol applied to round spermatids: phROSI. (**a**) Representation of phROSI showing (top left) Hoffman modulation image of round spermatid pick-up (bar, 5 μm). (**b**) Histograms showing *in vitro* development at E5.0 following phICSI (left) and phROSI of embryos injected at the times shown (0–2 h) after pronuclear membrane breakdown (pnMBD). Data are for all embryos (open) or those expressing paternal membrane tdTomato (mtdT). Values of *n* are from experiments on 2 days, with differences shown for *P*<0.05 (*χ*^2^ test). (**c**) Vertically paired Hoffman (upper) and fluorescence (mtdT) images of blastocysts (E5.0) developing from parthenogenetic haploid embryos (1nP) and phROSI-pnMBD-2h. Scale bar, 100 μm. (**d**) Average term (P0) weights of pups (left) and placentae. For ROSI, *n*=16; phROSI, *n*=6. Experiments were on 3 days. Differences are shown where *P*<0.05 (two-tailed, unpaired *t*-test). (**e**) Representative images of term (P0) pups and placentae (left panels) and F1 phROSI-pnMBD-2h and control ROSI offspring following natural mating. Values in **b** and **d** are ±s.e.m. and show *P*<0.05.

We next evaluated whether parthenogenotes were able to reprogram nucleosomal chromatin. Round spermatids were considered good candidates because naive pluripotent ES cells have been obtained from blastocysts produced by round spermatid injection (ROSI) into ph 2-cell embryos^14^. Accordingly, we performed ROSI into ph embryos (phROSI) within 30 min of pnMBD (phROSI-pnMBD-0 h) or 1 or 2 h post-pnMBD (Fig. 3a). Ubiquitously expressed transgenic *mtdT* was used to mark the paternal (spermatid) participation in development. Although development in phICSI declined the greater the time between pnMBD and sperm injection (*n*≥73), the highest developmental rate for phROSI was when round spermatid nuclei were injected 2.0–2.5 h post-pnMBD (phROSI-pnMBD-2 h; 34.1%; *n*=123), with 26.8% mtdT-positive blastocysts (Fig. 3b,c; Supplementary Fig. 7a,b). These results show that embryos develop *in vitro* following phROSI, with paternal genome participation, and that optimal paternal chromatin reprogramming windows are different for nucleosomal round spermatids (phROSI)^23^ and protaminic sperm (phICSI).

To examine developmental potential following phROSI, phROSI-pnMBD-2 h 2-cell embryos were transferred to surrogate mothers (Fig. 3d,e). Live pups were produced at a rate of 2.6% per transferred embryo (*n*=213), with 31.7% of transferred control ROSI embryos (*n*=50) producing live offspring. ROSI and phROSI average body masses were similar at term (1.38±0.05 g (*n*=16) versus 1.49±0.09 g (*n*=6), ROSI versus phROSI) but phROSI placentas were larger (0.14±0.01 g (*n*=16) versus 0.17±0.02 g (*n*=6), *P*=0.028; Fig. 3d), possibly due to the larger average litter sizes in ROSI (3.2 versus 1.2). Both ROSI and phROSI offspring could develop into fertile adulthood (Fig. 3e). These results indicate that the mitotic machinery of 1-cell stage embryos can directly remodel round spermatid nucleosomal chromatin leading to the production of healthy offspring.

### Developmental and epigenetic reprogramming in phICSI

In phICSI, sperm reprogramming bypasses early embryonic events (for example, meiotic exit, DNA synthesis and pn formation) and is no longer synchronised with maternal chromatin remodelling. We took developmental and epigenetic approaches to investigate whether this might account for depressed rates of phICSI-13 development compared with those of ICSI controls.

**Figure 4:**
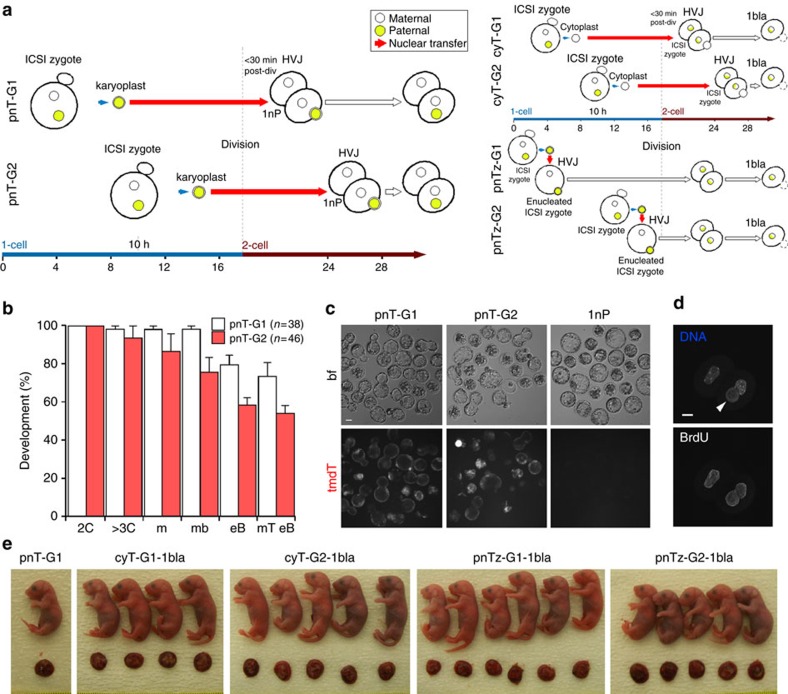
Male pronuclear transfer to haploid parthenogenotes. (**a**) Schematic representations of male pronuclear transfer into one blastomere of 2-cell haploid parthenogenotes (pnT-G1 and -G2, left). Schematic representations of control zygote cytoplast transfer into a 2-cell haploid parthenogenote blastomere (cytT-G1 and -G2, upper right) and pronuclear transfer into paternally-enucleated zygote (pnTz-G1 and -G2, lower right) are also shown. HVJ, hemagglutinating virus of Japan envelope vector. These controls determine *in vivo* developmental potential following manipulation at the 2-cell stage; data are presented in Supplementary Table 1. (**b**) Histograms showing *in vitro* developmental progress of pnT-G1 (open) and -G2 embryos (± s.e.m.). For pnT-G1, *n*=38 and pnT-G2, *n*=46, with experiments on 2 days. (**c**) Vertically paired images representative of *in vitro* development for pnT-G1 and -G2 E5.0 embryos and haploid parthenogenotes (1nP) showing Hoffman (bf, top) and paternally expressed membrane tdTomato (mtdT). Scale bar, 50 μm. (**d**) Paired fluorescence images of pnT-G1 embryos at the 2-cell stage showing DNA labelling (left) or treatment with BrdU (*n*=7). (**e**) Pups and associated placentae in pnT-G1 and the indicated control experiments, as they appeared after term delivery 19 d after embryo transfer (P0).

As a control for developmental stage skipping in early embryogenesis, including the possible dysregulation of DNA synthesis, we transferred male pn (containing the *mtdT* transgene) of ICSI-derived embryos into ph 2-cell blastomeres. We based transfer timing on early embryo (pro)nuclear membrane breakdown and division kinetics (Supplementary Fig. 8a–c), and on BrdU incorporation as an indicator of S-phase DNA synthesis (Supplementary Fig. 8d,e). Times of corresponding (pro)nuclear membrane breakdown and cell division events were respectively similar in ph and ICSI-derived embryos (Supplementary Fig. 8a–c). BrdU incorporation in the nuclei of 81.8% (*n*=11) of 2-cell ph embryos had already initiated 1–2 h after 1- to 2-cell cleavage (Supplementary Fig. 8d,e). From these data, we ascribed zygotes at 4 h and new 2-cell embryos (<30 min post division) to an early, G1-phase group (pnT-G1) and zygotes at 10 h and later 2-cell embryos (28 h after ICSI or SrCl_2_ treatment) to a late, G2-phase group (pnT-G2; Fig. 4a).

When pn were transferred 4 h post-ICSI (with *mtdT* sperm) into haploid blastomeres <30 min after the first mitotic division (pnT-G1), 79.8±4.8% (*n*=38) of the embryos developed to the expanded blastocyst stage and 73.7±9.0% were mtdT-positive (Fig. 4b,c; Supplementary Table 1). DNA synthesis was detected in all pnT-G1 embryos after pn transfer (*n*=7; Fig. 4d). Transfer of late-stage pn (10 h after *mtdT* ICSI) to 2-cell ph blastomeres 28 h after SrCl_2_ exposure (pnT-G2, *n*=46), produced 58.5±4.8% blastocysts, of which 92.6±7.2% were mtdT-positive (Fig. 4a–c). When 2-cell embryos from the pnT-G1 group were transferred to surrogate mothers, two (3.6±0.1%, *n*=56) developed to term, although both died perinatally^15^ and there were no offspring in the pnT-G2 group (Fig. 4e; Supplementary Table 1). Control pronuclear (pnTz-G1 and -G2) and cytoplast transfer (CyT-G1 and -G2) (Fig. 4a) resulted rates of term (31.9–46.3%, 35≤*n*≤39; Fig. 4e) that were comparable to those of ICSI-1bla embryos (Supplementary Table 1).

These results are consistent with multiple steps being required for sperm chromatin to attain high developmental potential during the change from meiotic-to-mitotic cell cycles. Some of these steps are omitted in phICSI and pronuclear transfer and may reduce reprogramming, although we cannot exclude a technical contribution including lack of precise cell cycle synchrony between donor pronuclei and recipients in the pnT series.

**Figure 5:**
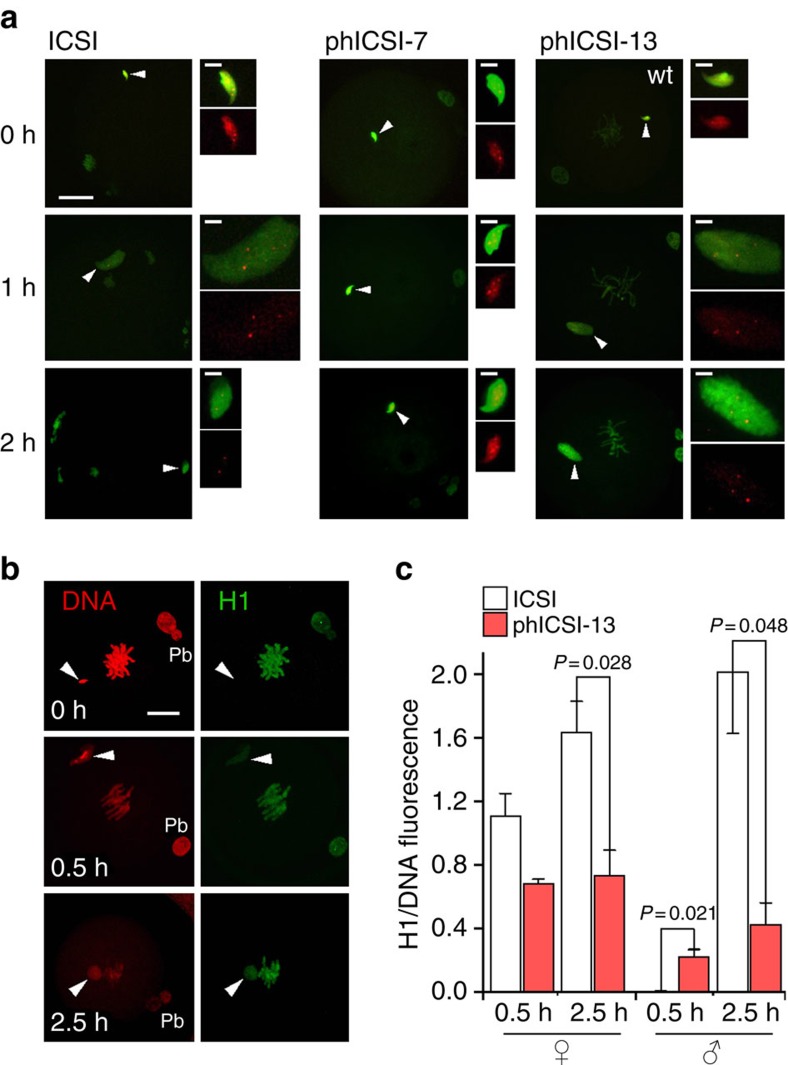
Nucleoprotein exchange in phICSI-13. (**a**) Merged fluorescence images showing protamine 2-mCherry (Prm2-mCh; anti-RFP, red) and genomic DNA (DNA; pico green, green) 0 (*n*=10), 1 (*n*=11) or 2 h (*n*=7) after *Prm2-mCh* transgenic sperm injection into mII oocytes, or parthenogenotes 7 h (for 0 h, *n*=7; 1 h, *n*=9; 2 h, *n*=9) or 13 h (for 0 h, *n*=13; 1 h, *n*=14; 2 h, *n*=14) after initial SrCl_2_ exposure (phICSI-7 and -13 respectively). White arrowheads show the positions of sperm heads. Scale bars, 20 μm; wt, wild-type. Close-ups of paternal chromatin (insets) are the same scale (bar, 5 μm). (**b**) Fluorescence images of DNA (propidium iodide, PI, left) and linker histone H1/H1foo at the times shown after sperm injection in phICSI-13. Pb, second polar body. Scale bars, 20 μm. (**c**) Pixel quantification (±s.e.m.; for ICSI, *n*=3 and phICSI-13, *n*=4, with experiments on 2 days) showing H1 fluorescence levels normalized against PI (DNA) associated with each parental genome at the times indicated after sperm injection in ICSI and phICSI. Significantly different (*P*<0.05) pair-wise comparisons are indicated.

We next characterized genome reprogramming in phICSI by comparing chromatin dynamics in phICSI and control ICSI embryos at the same times after sperm injection. Most protamine was lost from transgenic protamine-mCherry (Prm2-mCherry) sperm within 2 h of ICSI (*n*=18) or phICSI-13 (8≤*n*≤14), but not phICSI-7 (7≤*n*≤9; Fig. 5a). Parental chromatin sets possessed 2- to 5-fold reductions of histone H1 levels 2.5 h after sperm injection in phICSI-13 (Fig. 5b,c; Supplementary Fig. 2d,e). Sperm dimensions remained unchanged in phICSI-7 for 5 h (Fig. 5a; Supplementary Fig. 2d) indicating decondensation failure. The paternal:maternal nuclear volume ratio in phICSI-13 embryos (0.8±0.1) was 66.7% of that in zygotes (1.2±0.1, *n*≥10, *P*<0.0001; Supplementary Fig. 9a), suggesting that anomalous nuclear remodelling occurred in phICSI-13 and allowing us to identify the paternal nucleus. Deposition of cRNA-encoded histone H3.3 onto paternal chromatin was slower in phICSI-13 than it was in control ICSI and had not been completed by the end of M-phase (Supplementary Fig. 10a). Taken with data from phICSI-7 (Fig. 5a; Supplementary Fig. 2d), this corroborates a higher efficiency of sperm chromatin remodelling during M-phase than other cell cycle stages^21^.

**Figure 6:**
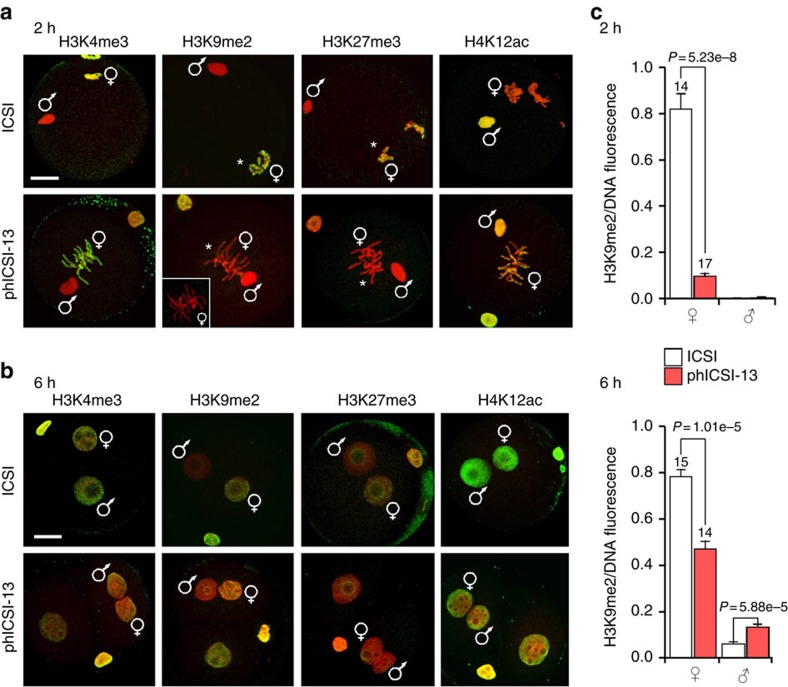
Conserved and divergent histone modification in ICSI and phICSI-13. (**a**) Merged confocal fluorescence images showing DNA (stained with propidium iodide, red) and the indicated histone modifications (detected using antibodies, green) 2 h after sperm injection in ICSI (upper panels) or phICSI-13. The parental provenance of chromatin is indicated. The inset shows H3K9me2 staining in ph-13 parthenogenotes. Asterisks indicate modification differences. Scale bar, 20 μm. For ICSI H3K4me3, *n*=11; phICSI-13 H3K4me3, *n*=16; ICSI H3K9me2, *n*=20; phICSI-13 H3K9me2, *n*=24; ICSI H3K27me3, *n*=18; phICSI-13 H3K27me3, *n*=14; ICSI H4K12ac, *n*=18; phICSI-13 H4K12ac, *n*=16. (**b**) As for (**a**), except 6 h after sperm injection. For ICSI H3K4me3, *n*=9; phICSI-13 H3K4me3, *n*=10; ICSI H3K9me2, *n*=20; phICSI-13 H3K9me2, *n*=22; ICSI H3K27me3, *n*=12; phICSI-13 H3K27me3, *n*=22; ICSI H4K12ac, *n*=14; phICSI-13 H4K12ac, *n*=24. (**c**) Histograms showing H3K9me2 fluorescence quantification in ICSI and phICSI, indicating *n* for each group. These are, for ICSI 2 h, *n*=14; ICSI 6 h, *n*=15; phICSI-13 2 h, *n*=17; phICSI-13 6 h, *n*=14. Values (± s.e.m.) indicate differences where *P*<0.05 (two-tailed, unpaired *t*-test). Experiments were on 3 days.

We next applied immunofluorescence microscopy^7^,^8^,^24^,^25^,^26^,^27^ to ask if epigenetic modifications also differed between phICSI and ICSI at given times after sperm injection. Global levels of H4K12ac in phICSI-13 were similar to controls 2 and 6 h after sperm injection (18≤*n*≤24; Fig. 6a,b). Although all phICSI-13 maternal H3K4me3 and H3K27me3 levels were initially low, by 6 h they were similar to ICSI (9≤*n*≤22; Fig. 6a,b). However, levels of H3K9me2 in phICSI-13 maternal chromatin were invariably persistently lower than controls (<85% levels at 2 h, <40% at 6 h; 20≤*n*≤24; Fig. 6a–c). These experiments compare given times after sperm injection and may not account for cell cycle or other potential disparities, but they indicate that the epigenetic trajectories taken for chromatin ICSI and phICSI exhibit similarities (for example, H4K12ac levels), differences that recovered (for example, H3K4me3 and H3K27me3 levels) and sustained disparities (for example, H3K9me2 levels).

**Figure 7:**
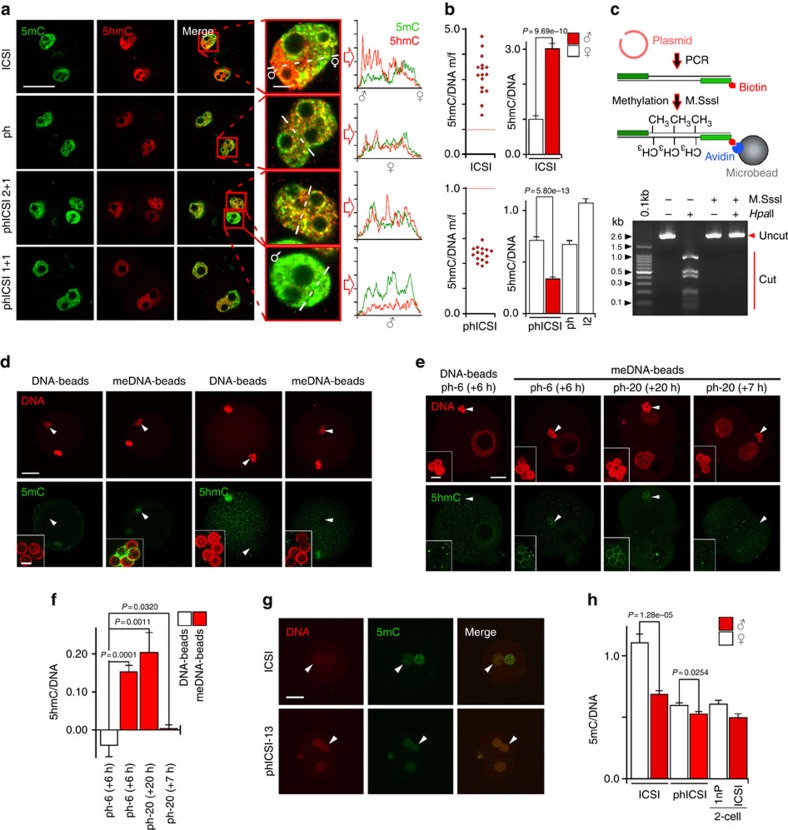
Altered genomic 5′-methylcytosine and 5′-hydroxymethylcytosine dynamics in phICSI-13. (**a**) Immunofluorescence showing nuclear 5′-methylcytosine (5mC, green) and 5′-hydroxymethylcytosine (5hmC, red) 30 h after the start of SrCl_2_ treatment or sperm injection (ICSI). Parthenogenetic haploid, ph; bar, 50 μm. Insets (bar, 2 μm) show merged nuclear images with line plots of relative intensities (rightmost). (**b**) Ratios (±s.e.m.) of relative paternal and maternal 5hmC fluorescence in individual (left) or pooled (histograms) 10 h after ICSI (upper; *n*=16), or 30 h after ICSI (2-cell embryos, I2; *n*=12) or the start of SrCl_2_ treatment in either parthenogenesis (ph; *n*=14) or phICSI-13 (phICSI; *n*=15). Experiments were on 2 days and differences are indicated where *P*<0.05 (two-tailed, unpaired *t*-test). (**c**) Protocol for generating microbeads conjugated to 5mC-containing DNA (5mC-beads), showing (below) electrophoresis of 5mC-containing DNA following treatment with the CpG DNA methyltransferase, M.SssI (M.SssI+) or unmethylated DNA (M.SssI-), challenged with the methylation-sensitive restriction endonuclease, *Hpa*II. (**d**) Fluorescence images showing 5mC or 5hmC (green) in beads conjugated to control, non-methylated DNA (DNA) or to 5mC-containing DNA (meDNA) 7 h after injection into in mII oocytes (scale bar, 20 μm), with insets showing close-ups (bar, 3 μm). DNA is stained with propidium iodide (PI). For DNA 5mC, *n*=7; meDNA 5mC, *n*=7; DNA 5hmC, *n*=7; meDNA 5hmC, *n*=10. (**e**) Fluorescence images of haploid parthenogenotes containing latex microbeads conjugated to non-methylated DNA (DNA-beads; *n*=7) or 5mC-containing DNA (meDNA-beads; *n*=7), stained with PI (DNA, red) or anti-5hmC antibodies (green). Beads were either injected into mII oocytes (mII) followed by Sr^2+^ activation and analysis 6 h (ph-6, 1-cell; *n*=10) or 20 h (ph-20, 2-cell; *n*=8) later, or ph-13 parthenogenotes were injected with beads followed by analysis at 20 h (+7 h) (*n*=7). Scale bar, 50 μm. Arrowheads indicate DNA-beads (magnified in insets; bar, 3 μm). (**f**) Fluorescence intensity quantification of 7≤*n*≤10 embryos of **e** showing *P*-values following one-way ANOVA followed by a Tukey–Kramer test. (**g**) Fluorescence staining with PI (DNA, red) or anti-5mC antibodies (5mC, green) showing relative paternal and maternal 5mC in ICSI and phICSI-13 embryos 6 h after sperm injection. Scale bar, 20 μm. (**h**) Pixel quantification (±s.e.m.) of samples (for ICSI, *n*=12; phICSI, *n*=11) of **g** and 1nP (*n*=10) and ICSI 2-cell embryos (*n*=14) 6 h post-cleavage, showing fluorescence levels normalized against PI (DNA). Experiments were on 2 days and differences are indicated where *P*<0.05 (two-tailed, unpaired *t*-test).

Since DNA modification is linked to histone remodelling and is a characteristic of zygotic reprogramming^28^, we investigated its behaviour in phICSI. Paternal 5hmC levels were higher (>100%) than maternal 5hmC levels in ICSI controls^7^ but in all (*n*=13) of 2+1-class phICSI-13 embryos the paternal:maternal ratio was markedly lower, at 55% (*P*=0.0055; Supplementary Fig. 9b,c). This pattern was always (*n*=10) conserved when sperm were injected into zygotes (zygotic ICSI, zICSI) and in phICSI-13 1+1 embryos (Supplementary Figs 9d,e and 11a,b). The low paternal:maternal 5hmC ratio always (*n*=15) persisted 14 h post division (48%; *P*<0.001; Fig. 7a,b) and to at least the second M-phase in all (*n*=18) embryos (Supplementary Fig. 11c).

Immunofluorescence analysis of global DNA modification in early embryos^7^,^8^,^24^,^25^,^26^,^27^ is supported by bisulphite sequencing^7^,^8^,^24^,^26^ requiring pooled samples. We wished to corroborate our findings at the single-cell level. To do so, we adapted an assay in which latex microbeads conjugated to DNA (DNA-beads)^29^,^30^,^31^,^32^ were injected into mII oocytes and 1-cell parthenogenotes (assay details are described in the Methods). In contrast to negative controls (beads lacking DNA), DNA-beads injected into mII oocytes stained with a DNA dye and after 6 h acquired H4K12ac and microtubules (Supplementary Fig. 12a–c). Deposition of cRNA-encoded histone H3.3 occurred at similar rates onto paternal chromatin in ICSI and DNA-beads (Supplementary Fig. 10a,b). These findings, corroborated below, suggest that the DNA-beads assay provided a faithful proxy for epigenetic modification in 1-cell embryos. We therefore further characterized 5hmC dynamics with latex beads conjugated to 5mC-containing DNA (5mC-beads; Fig. 7c,d; Supplementary Fig. 12b,c). Beads injected into mII oocytes were labelled by anti-5mC antibody after 7 h, but 5hmC was undetected (Fig. 7d; Supplementary Fig. 12d). 5hmC was also undetected after ∼7 h on 5mC-beads injected into ph-13 parthenogenotes (ph-20 (+7 h) in Fig. 7e). However, when oocytes injected with 5mC-beads were activated with SrCl_2_, 5hmC became detectable after 6–7 h (ph-6 in Fig. 7e and 7h in Supplementary Fig. 12e) and remained after 20–30 h (ph-20 (+20 h) in Fig. 7e and 30 h in Supplementary Fig. 12e). 5hmC was not detectable when the beads were conjugated to unmethylated DNA (Fig. 7e). This suggests that early embryonic regulation of 5hmC is 5mC- and cell-cycle-dependent, consistent with differences between ICSI and phICSI-13 (Fig. 7a,b; Supplementary Fig. 9b,c).

Given that the dynamics of 5hmC were abnormal in phICSI-13, we determined whether the kinetics of 5mC were also disrupted. Control embryos (*n*=12) contained low paternal:maternal genomic 5mC 6 h after ICSI as previously noted^4^,^6^,^7^, but phICSI-13 embryos at the same time after sperm injection all (*n*=12) possessed a higher ratio, reflecting lower relative maternal 5mC levels (Fig. 7g,h; for parental ratios in ICSI versus phICSI, *P*=2.22e−05). These findings argue that early embryonic 5mC and 5hmC regulation is coupled and that some embryos are tolerant of phICSI-associated dysregulation of the signature DNA modification dynamics of fertilization that precede normal development.

### The early transcriptome in phICSI is abnormal

To evaluate whether altered epigenetic regulation in phICSI affected gene expression^33^, we performed comparative microarray analyses of phICSI-13 2-cell biparental blastomeres and ICSI controls (Supplementary Table 1) in which major embryonic gene activation has normally occurred^34^,^35^.

**Figure 8:**
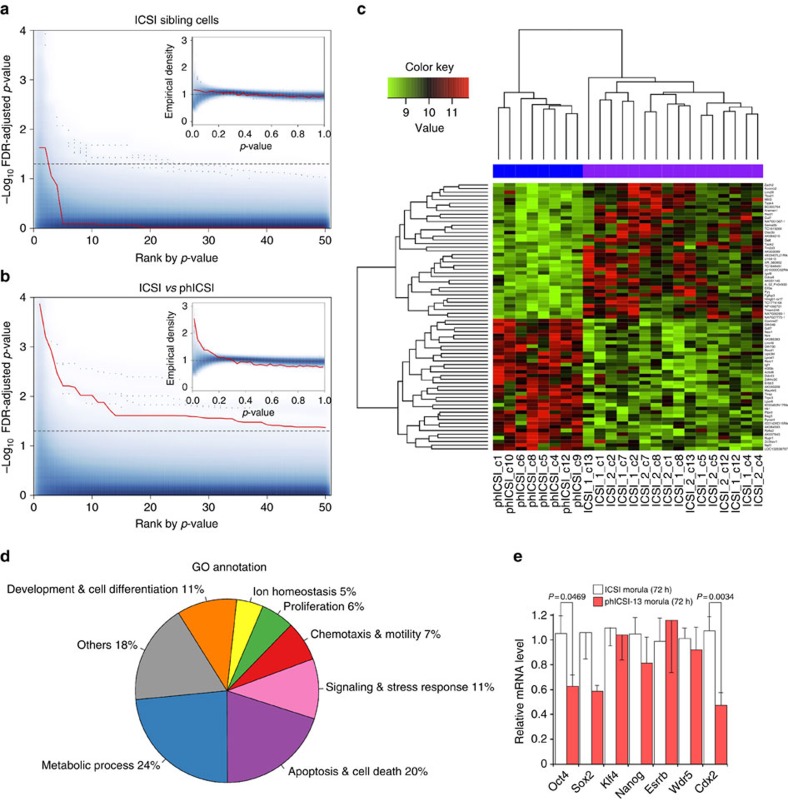
Distinctive ICSI and phICSI 2-cell embryo transcriptomes. (**a**) FDR-adjusted *P*-values for all 6,435 possible array assignments to the two equally-sized (8+8) ICSI groups (smoothed blue area) plotted over the gene rank according to smallest *P*-value. The red curve indicates the most extreme (largest number of adjusted *P*-values <0.05) combination that respects the experimental 2-cell sibling pairings. The inset shows the empirical density of *P*-values. Where there is no difference between ICSI and phICSI (true null hypothesis), a uniform density is expected. (**b**) FDR-adjusted *P*-values calculated for 10,000 out of 735,471 possible 16+8 ICSI-phICSI array groupings (smoothed blue area). The red curve represents the true ICSI-phICSI group assignment. (**c**) Normalized heatmap for the top 73 most differentially expressed genes (FDR *P*<0.05) between ICSI and phICSI. Values indicate different blastomeres. ICSI_1 and ICSI_2 refer to paired sibling cells in ICSI 2-cell embryos. (**d**) Classes of GO annotation terms obtained from the DAVID database. (**e**) Ratiometric qPCR analysis of pluripotency factor mRNAs in ICSI-1bla (*n*=5) and phICSI-13-1bla (*n*=8) morulae after 72 h. Values are±s.e.m. and differences indicated where *P*<0.05 (unpaired *t*-test).

Consistent with our previous findings^36^, sibling cells from ICSI 2-cell embryos exhibited very similar gene expression profiles: only 4 out of the 128 possible combinations respecting the experimental 2-cell pairing exhibited a single gene with a false discovery rate- (FDR-) adjusted *P*-value <0.05 and only one combination resulted in a maximum of two significant genes. Unadjusted *P*-values were consistently uniformly distributed (Fig. 8a). By contrast, gene expression between phICSI-13 and ICSI 2-cell blastomeres was clearly distinct (Fig. 8b). Transcriptome differences (FDR-adjusted *P*<0.05) between ICSI and phICSI-13 blastomeres were evident for 73 genes (Fig. 8b,c). Gene Ontology (GO) annotation retrieved from the DAVID database showed that gene expression differed between phICSI and ICSI regarding ion homeostasis, cell proliferation and development (Fig. 8d). None of these associations were significant as judged by Fisher's Exact Test on the 5% level (lowest, *P*=0.206 for cation homeostasis). Analogous pathway analysis using DAVID gave significant enrichment for N-glycan biosynthesis, monosaccharide metabolism, toll-like receptor pathways (all apparently up-regulated in phICSI-13) and IL-3 signalling pathways (apparently down-regulated in phICSI-13; lowest *P*=0.108, for IL-3). Notwithstanding transcriptomic disparities between control and phICSI-13 two-cell embryos, 61.6% of phICSI-13-1bla embryos reached the morula stage before developmental failure (Supplementary Table 1), prompting us to examine the regulation of pluripotency factor genes around the time of morula arrest. Transcript levels were reduced for *Oct4* (∼40%, *P*=0.0469) and *Cdx2* (∼50%, *P*=0.0034) at the morula stage (Fig. 8e). This coincides with lower transgenic *Oct4* promoter-driven mCherry expression (Supplementary Fig. 4e), although the balance of Oct4/Cdx2 protein expression had normalized with respect to controls in phICSI-13 embryos reaching the blastocyst stage (48 h later)(Supplementary Fig. 4f). Thus, dysregulation of pluripotency factor expression may contribute to developmental arrest in phICSI embryos during preimplantation lineage specification.

## Discussion

These studies demonstrate that mitotic embryos are able to remodel sperm chromatin completely, leading to the direct production of healthy animals. Full sperm reprogramming is therefore not unique to oocytes, showing that sperm chromatin reprogramming machinery is present at different development stages and in other cell types. phICSI has the potential to reveal not only stage-dependent features such as high zygotic 5hmC production, but key features of reprogramming during the gamete-to-embryo transition in natural fertilization. All randomly-sampled phICSI embryos exhibited anomalous reprogramming, but since full-term development in phICSI-13-1bla occurred at 24% of ICSI control rates (10.4% versus 43.4%, Supplementary Table 1), development was not the result of a subgroup that evaded epigenetic analyses. We challenged this assertion by generating binomial distributions of probabilities that phICSI embryos with two normal modifications gave rise to different numbers of offspring (Supplementary Fig. 13). The production of 9 or more phICSI embryos with modifications characteristic of ICSI out of a total of 24 that developed to term can be excluded on the 5%-significance level, since *P*(9|20,*ρ*)≤0.0465 (worst case) for all *ρ* (Supplementary Fig. 13). The probability that not a single embryo is phICSI modification derived is only *P*(24|20, *ρ*)≤9.84e−17. Although this analysis assumed only two epigenetic differences, we detected five modifications that consistently differed between stage-matched ICSI and phICSI-13 embryos. These data strongly suggest that different chromatin remodelling dynamics support the emergence of totipotency following phICSI.

Given that sperm reprogramming naturally accompanies exit from the second meiotic metaphase, it is reasonable to suppose that our success in phICSI-13 was partly because recipient parthenogenotes were at, or soon entered, metaphase of the first mitotic M-phase after sperm injection^22^. This is consistent with the lower rates of term development we observed in phICSI-7 and -10, where sperm were injected during the meiotic-to-mitotic transition well before M-phase (Supplementary Table 1). This constitutes evidence that a cell cycle window for sperm reprogramming closes after fertilization and reopens during entry into the first mitotic M-phase.

An additional possibility is that sperm reprogramming factors accumulate in pronuclei during interphase^37^. Pronuclear sequestration of remodelling factors is also thought to occur in somatic cell nuclear transfer, but the mitotic cytoplasm later regains remodelling activity^15^,^38^. Injected sperm heads in phICSI-7 and -10 exhibited limited nucleoprotein exchange until pn membrane breakdown (Supplementary Fig. 2) and poor subsequent development may have been due to paternal chromatin damage before M-phase (Supplementary Table 1).

When we employed pn transfer experiments to ask whether embryonic stage skipping exerted an effect on development, pups were produced only in pre-S-phase transfer experiments (the pnT-G1 group) but they did not survive fostering (Fig. 4a–c; Supplementary Table 1). We cannot exclude technical effects such as genomic DNA damage and cell cycle discordance between donor pn and recipient blastomeres, but it is also possible that key reprogramming events of the first mitotic cell cycle were omitted in both pnT-G1 and pnT-G2 and that these events are part of the physiological mechanism of the gamete-to-embryo transition following natural fertilization.

From the unique properties of sperm chromatin^3^ it might be inferred that remodelling activity in oocytes is unique, but in phICSI, remodelling is performed by mitotic embryos. This implies that nucleoplasmin-like activity responsible for removing protamines is also mitotic^5^. Mouse putative nucleoplasmins Npm1, Npm2 and Npm3 are expressed in oocytes; after fertilization, Npm2 is present in both parental pronuclei and remains in the nuclei of early cleavage embryos^39^. Pronuclear sequestration, embryonic persistence and evidence that Npm2 possesses somatic cell reprogramming activity^40^ collectively suggest that one or more nucleoplasmins mediate chromatin remodelling in phICSI.

In fertilization, sperm decondensation is followed by maternal histone deposition^3^. Deposition of the oocyte core histone variant, H3.3 requires the chaperone, Hira^41^ and since Hira is present in mitosis^27^ it may operate in mitotic sperm chromatin remodelling. Chromatin remodelling in fertilization and mitotic cells may share other features including rapid and dynamic regulation of nuclear proteins, H3 family dynamics and the contribution of factors enriched in mII oocytes^41^,^42^,^43^,^44^. The success of phROSI shows that mitotic parthenogenotes can directly reprogram non-protaminic chromatin from differentiated cells; the method might also be combined with products of paternal meiosis generated *in vitro*^45^.

High developmental rates in phICSI are remarkable given that the injected sperm bypasses all events as they occur during the first 13 h of natural fertilization (Supplementary Fig. 1a), including calcium mobilization, cytokinesis, disassembly of membrane, mitochondria and perinuclear structures, chromatin decondensation, reassembly and modification leading to pn formation, S-phase and preparation for the first embryonic transcription^2^,^5^. However, an essential reprogramming role for calcium mobilization is unlikely; it is dispensable for full development and is probably more important for sustained Emi2 degradation^1^,^46^. Fertilizing sperm desconstruction inside the oocyte is mediated by the proteasome, which is ubiquitously expressed and present in early mammalian embryos^47^,^48^. This is consistent with evident dismantling of sperm in phICSI-13, although we did not examine sperm dismantling in detail in phICSI-7 and -10; the prediction is that although sperm chromatin remained condensed in phICSI-7 and -10 (Fig. 5a), some components disperse. The oocyte microvillar projections thought to remove the sperm perinuclear matrix following fertilization are rich in microfilaments^49^. Since microfilaments persist well into the late 1-cell stage^50^, analogous interactions could remove the perinuclear matrix in successful phICSI.

Chromatin remodelling following fertilization involves pronounced changes in 5hmC levels^8^ which were markedly different in phICSI (Fig. 7a,b). Hydroxymethylation of paternal chromatin or of 5mC DNA-beads (5mC-beads) injected around the first M-phase was low compared to ICSI controls (Fig. 7b). Under the conditions of the DNA-beads assay, the appearance of 5hmC required pre-existing 5mC. Given that rates of phICSI-13 term development (Supplementary Table 1) were similar to those of functionally *Tet3 null* embryos^7^, it is possible that 5hmC dysregulation accounts for some developmental failure in phICSI but it is unlikely to account for all of it. First, if 5hmC anomalies alone accounted for impaired phICSI development, other aspects of reprogramming in phICSI would either be the same as those of ICSI, which they are not, or the 5hmC differences in phICSI would have to have been indirectly responsible for all of the epigenetic differences we observed. Secondly, lack of functional maternal Tet3 does not affect rates of preimplantation development^7^, but preimplantation development in phICSI-13 was poor (Supplementary Table 1). Thirdly, during manuscript preparation, it was reported that haploinsufficiency is the major reason for the perinatal abnormality in Tet3 mutant mice, with no fundamental developmental function of Tet3-mediated 5hmC production in male pronuclei^51^. Fourthly, 5mC-dependent DNA hydroxymethylation is evident in ph-7 parthenogenotes (Fig. 7e; Supplementary Fig. 12b,c), suggesting that phICSI and embryos lacking functional *Tet3* are not equivalent. Finally, zygotic Dppa3/Pgc7/Stella binds H3K9me2 to prevent Tet3-mediated conversion of 5mC to 5hmC, at least in imprinted genes^27^, but in phICSI, the dynamics of 5hmC and H3K9me2 were disrupted.

The ability of mitotic embryos to reprogram sperm in phICSI blurs functional distinctions between somatic, embryonic and gametic cell lineages. This finding could benefit mammalian embryology when parthenogenotes are available but oocytes are not^52^ and it is perhaps poignant that we generated phICSI offspring with both chemically (Sr^2+^-) and spontaneously (Plcξ-) activated parthenogenotes. Moreover, our work calls into question the argument that parthenogenotes do not have the potential for full-term development and are accordingly a more acceptable source of human ES cells^53^,^54^. The implication that the totipotent state may be arrived at via different epigenetic kinetics or pathways hints that for other cell types different routes might also exist to a given new cellular potency destination.

## Methods

### Collection and culture of oocytes

Animal procedures complied with the Animals (Scientific Procedures) Act, 1986. Wild-type mouse (*Mus musculus*) strains were bred from C57BL/6 or DBA/2 stocks in-house or otherwise supplied by Charles River (L'Arbresle, France). Mixed C57BL/6 and B6D2F1 background hybrid lines containing the transgenes *pOct4-mCherry*, *pPrm2-Prm2-mCherry*, *pCAG-Plcz-ires-Venus*^20^, *Gt(ROSA)26Sor*^*tm4(ACTB-tdTomato,-EGFP)Luo*^(mT/mG)^22^, *pPGK-Cre* or *pNanog-eGFP*^55^ were generated in-house or were kind gifts. Oocytes were collected from 8- to 12-week-old females that had been super-ovulated by standard serial intraperitoneal injection of 5 IU pregnant mare serum gonadotropin (PMSG) followed 48 h later by 5 IU human chorionic gonadotropin (hCG)^56^. Oviductal metaphase II (mII) oocytes were collected in M2 medium (EMD Millipore, UK)^57^ ∼15 h post-hCG injection and cumulus cells dispersed by hyaluronidase treatment^56^. After repeated washing in M2, denuded oocytes were incubated in kalium simplex optimized medium (KSOM; Millipore)^58^ under mineral oil in humidified 5% CO_2_ (v/v air) at 37 °C, until required. Where appropriate (for example, for diagnosis of fertilization by second polar body extrusion), mII oocytes with a degenerate first polar body were selected; by 16 h post-hCG, 71.0±2.0% (*n*=2,294) of first polar bodies had degenerated. Activation of B6D2F1 or mT/mG oocytes to produce parthenogenetic haploid embryos was in Ca^2+^-free CZB medium supplemented with 10 mM SrCl_2_, for 2.5 h in humidified 5% CO_2_ (v/v air) at 37 °C (refs 59, 60). SrCl_2_ treatment was initiated 16–17.5 h post-hCG; times are given after the start of SrCl_2_ treatment. For phICSI and controls, embryos were chosen that possessed a second polar body and a single pn at 5–7 h. For phICSI, SrCl_2_-activated haploid parthenogenotes were injected with sperm at the times indicated after commencement of SrCl_2_ treatment. Spontaneously arising parthenogenotes containing a single pn were selected from *pCAG-Plcz-ires-Venus* females^20^ following brief culture *in vitro*. For maternal genome labelling with 5-bromo-2'-deoxyuridine (BrdU), haploid B6D2F1 parthenogenotes were cultured in KSOM containing 5 μM BrdU (Sigma) for 5 h, starting 4 h after SrCl_2_ treatment was initiated. S-phase was determined by culturing 2-cell ph embryos for 1 h at the appropriate times in KSOM containing 100 μM BrdU.

### Sperm preparation and microinjection

For sperm preparation, cauda epididymidal sperm from 8- to 12-week-old males were triturated for 45 s in nuclear isolation medium (NIM; 125 mM KCl, 2.6 mM NaCl, 7.8 mM Na_2_HPO_4_, 1.4 mM KH_2_PO_4_, 3.0 mM EDTA; pH-7.0) containing 1.0% (w/v) 3-[(3-cholamidopropyl)dimethylammonio]-1-propanesulfonate (CHAPS) at room temperature (25 °C)^56^,^58^,^59^. Sperm were washed twice in NIM and pelleted (1,890 *g*) at ambient temperature; head-tail detachment was enhanced by trituration during pellet resuspension. Finally, sperm were resuspended in ice-cold NIM (∼0.5 ml per epididymis) and stored at 4 °C for up to 3 h until required. ∼50 μl of each suspension was typically mixed with 20 μl of polyvinylpyrrolidone (PVP, average *M*_r_≈360 000; Sigma-Aldrich) solution (15% (w/v)) and sperm injected (ICSI) into oocytes in droplet of M2 within ∼60 min, essentially as described^56^. Injected oocytes were transferred to KSOM under mineral oil equilibrated in humidified 5% CO_2_ (v/v air) at 37 °C. For phICSI, SrCl_2_-activated haploid parthenogenotes were injected with sperm at the times indicated after commencement of SrCl_2_ treatment. For phICSI-13, this was 13 h after the initiation of SrCl_2_ treatment of B6D2F1 oocytes, just after pnMBD. Where parthenogenotes were from *pCAG-Plcz-ires-Venus*, sperm were injected soon after pnMBD. In some experiments (Supplementary Fig. 9d,e), zygotes produced by mating were injected with a sperm immediately after pnMBD, referred to as zygotic ICSI, zICSI.

### Ablation of single 2-cell embryo blastomeres

One of the two blastomeres in 2-cell embryos was destroyed by suction of nuclear material into a piezo-actuated pipette (inner diameter, 7–8 μm). In the case of phICSI-13, embryos of the 2+1 nuclear type (Fig. 1b) were selected and the blastomere with a single (maternal) nucleus was destroyed.

### Pronuclear transfer and cytoplast transfer

For paternal pn and zygotic cytoplast transfer^60^,^61^, the zona pellucida of donor and recipient embryos was cut in M2 medium with a fine microneedle. Donor zygotes produced by ICSI (B6D2F1 oocytes and B6D2F1 or *mtdT* sperm) were held for 10–15 min in KSOM containing cytochalasin B (5 μg ml^−1^) and nocodazole (1 μg ml^−1^) just before manipulation in M2 medium similarly supplemented with cytochalasin B (5 μg ml^−1^) and nocodazole (1 μg ml^−1^). Part of the zygote containing the male pn was aspirated into a micropipette (outer diameter, 15 μm) and introduced with inactivated sendai virus (HVJ) envelope (GenomeONE-CF, Ishikawa Sangyo Kaisya, Japan) diluted 10x from a stock solution, into the space between the blastomere and zona pellucida of a 2-cell parthenogenetic haploid BDF1 embryo. Male pronuclear transfer into male pn-enucleated zygotes and zygotic cytoplast transfer into 2-cell blastomeres of biparental ICSI embryos were performed as controls.

### ROSI and phROSI

ROSI was performed by injecting B6D2F1 mII oocytes with round spermatid nuclei (from 8 to 10-week-old B6D2F1 or *mtdT*) in M2 medium, followed by activation with 10 mM SrCl_2_ in calcium-free CZB-G medium for 2.5 h (ref. 13). phROSI was performed by applying the ROSI procedure to parthenogenetic haploid embryos entering the first mitosis as nucleus recipients.

### Generation of recombinant fusion constructs

Recombinant constructs were used for the expression of cRNA and/or transgenes encoding fusion proteins. To generate the backbone vector, pCI-neo-mKO2-FLAG, an mKO2 *Sma*I/*Eco*RV fragment was generated by PCR from the Kusabira Orange-encoding plasmid, pmKO2-SI (Medical & Biological Laboratories, UK) and cloned into the mammalian expression vector, pCI-neo (Promega, UK). Into this construct, we inserted an *Eco*RV/*Not*I fragment derived from p3XFLAG-CMV-14 (Sigma-Aldrich, UK) to generate an mKO2-FLAG_3_ fusion. H3.3 was cloned into pCI-Neo-mKO2-FLAG as an *Xba*I/*Sal*GI fragment from cDNA generated by mII oocyte RT-PCR. The *Prm2* gene was cloned following PCR amplification of tail tip genomic DNA as an *Xho*I/*Eco*RI fragment that includes ∼1 kb upstream of the *Prm2* start codon, and inserted into pCI-neo-mCherry^1^. Expression of mVenus-hGeminin and mCherry-hCdt1 (the kind gifts of the RIKEN Brain Science Institute, Wako, Japan) was after they had been cloned as PCR-amplified *Eco*RI/*Xba*I fragments into pCI-neo. To generate the transgene construct in which the *Oct4A* promoter drives mCherry expression, a fragment including the *Oct4A* start codon and ∼5 kb of upstream promoter sequences^62^ was generated by PCR and cloned with an mCherry reporter fragment into the *Not*I site of vector pGEM-T (Promega).

### Embryo transfer

E1.5 (2-cell) embryos (the day following activation) were transferred to the oviductal ampullae of pseudo-pregnant CD-1 females at day 0.5 (that is, plugged females that had been mated with vasectomized males the previous night). Pups were delivered by natural birth and where appropriate, fetuses, pups and placentae collected by Caesarian section at the desired time point. Pups and placentas collected by caesarian section at term (E19.5) were weighed immediately. Newborn pups were fostered by CD-1 females.

### Genotyping

Mouse tissue samples were digested at 55 °C for 3 h in 25–100 μl of a lysis buffer containing 10% (w/v) sodium dodecyl sulfate and with 2 mg ml^−1^ proteinase K (Sigma). 1 μl of a 1:10 dilution of each sample was used for genotyping by PCR in a 10 μl reaction volume. PCR primer sequences are given in Supplementary Table 2. Primers for microsatellite analysis were selected from the Mouse Microsatellite Data Base of Japan^63^.

### Preparation and injection of cRNA

5'-capped and polyadenylated cRNA was synthesized from linearized plasmid template DNA in a T7 mScript Standard mRNA Production System (Cellscript, USA) according to the recommendations of the manufacturer^46^,^64^. cRNAs were dissolved in nuclease-free water, quantified on a Nanophotometer and stored in aliquots at −80 °C until required. cRNA solutions were diluted as appropriate with sterile water and injected (typically at concentrations of 0.01 to 1 μg μl^−1^) within 1 h of thawing via a piezo-actuated micropipette into mII oocytes or embryos in M2 medium. Following cRNA injection, oocytes or embryos were cultured for at least 3 h prior to subsequent manipulation.

### Immunocytochemistry

5′-Methylcytosine (5mC), hydroxymethylcytosine (5hmC) and BrdU were detected in embryonic DNA following fixation in 4% (w/v) paraformaldehyde and treatment with 2 M HCl for 30 min. Fixed embryos were processed as soon as possible but were stored where necessary at 4 °C. For primary antibody labelling, samples were incubated overnight at 4 °C with mouse anti-5mC antibody (1:200 (v/v); EMD Millipore, UK), for 1.5 h at 37 °C with rabbit anti-5hmC antibody (1:200; Active Motif, USA) or for 1.5 h with rat anti-BrdU antibody (1:100; Abcam, UK). Additional primary antibodies recognized Oct4 (1:100; Santa Cruz, USA), Cdx2 (1:100; BioGenex Laboratories, USA), H3K4me3 (1:250; Abcam, UK), H3K9me2 (1:50; Abcam), H3K27me3 (1:50; Abcam) and H4K12ac (1:250; Abcam). Primary antibody incubation was followed by a 1 h incubation at 37 °C with the appropriate secondary antibody (1:250; Life Technologies, UK) conjugated to Alexa 350, Alexa 488 and/or Alexa 594. DNA was stained by incubating samples at 37 °C for 20 min in propidium iodide (1:200; Sigma, USA) or Hoechst 33342 (1:1,000; Sigma).

### Fluorescence imaging

Images of live oocytes or embryos following cRNA injection were captured on an Olympus IX71 stand equipped with an Andro Zyla sCMOS camera and OptoLED illumination system (Cairn Research, UK) and processed using Metamorph software (Molecular Devices, LLC, USA). Excitation at 587 nm in combination with an ET-mCherry filter system was used for mCherry fluorescence detection and at 484 nm with an ET-EYFP filter system to detect Venus epifluorescence. Fluorescence of fixed samples was visualized on an Eclipse E600 (Nikon, Japan) microscope equipped with a Radiance 2100 laser scanning confocal system (BioRad, USA)^46^. Images were processed with ImageJ (imagej.nih.gov/ij/) or MetaMorph (Molecular Devices, USA) analysis software. Quantitative analyses subtracted background from subject area fluorescence intensities, which can produce negative results in beads experiments in which background levels from latex are lower than those of oocytes. Mouse fetus fluorescence stereomicrographs (Supplementary Fig. 6c) were captured by a Leica MZ16 FA fluorescence stereomicroscope, with LAS AF 4.0 imaging software (Leica Microsystems GmbH, Germany).

### Nuclear volume estimation

Nuclear volumes were estimated in zygotes and 2+1 type phICSI-13 2-cell embryos respectively 9 h post-ICSI or 30 h after initial SrCl_2_ exposure. These times correspond to approximately the same period after the paternal genome has completed its first S-phase respectively in ICSI and phICSI. Maternal nuclei were distinguished by comparatively low zygotic 5hmC or by BrdU labelling in phICSI. Propidium iodide staining was determined in z-stacked confocal images using ImageJ.

### Blastocyst cell counting

Blastocyst cell counting^46^ was performed by fixing blastocysts in 4% (w/v) paraformaldehyde and incubating them at 4 °C overnight in rabbit anti-Oct4 antibody (1:100; Santa Cruz) or for 1 h at 37 °C in mouse anti-Cdx2 antibody (1:100; BioGenex, USA), followed by 1 h at 37 °C in Alexa 488-conjugated anti-rabbit IgG (Invitrogen) or Alexa 594-conjugated anti-mouse IgG (Invitrogen) respectively. Cells stained with Alexa 488 were scored as Oct4-positive (pluriblasts) and those with Alexa 594, as Cdx2-positive (trophoblasts).

### DNA-conjugated latex microbeads

We adapted a method using streptavidin latex microbeads (Dynabeads; Invitrogen, MA, USA) conjugated to biotinylated DNA, so that we could perform high-resolution physiological analysis of chromatin and spindle function in living oocytes and embryos, as previously performed for spindles^30^,^31^, chromatin^29^,^30^,^31^ and mouse oocytes^32^. DNA fragments (2,605 bp) were amplified using LA Taq DNA polymerase (TAKARA BIO, Japan) from plasmid pCI-Neo (Promega Corp., WI, USA) using the primer pair (5'**→**3'): sense, CTGGCGTAATAGCGAAGAGG; antisense, ATAATACCGCGCCACATAGC. The sense primer was pre-labelled with biotin at its 5' end (Invitrogen). Following agarose gel electrophoresis, DNA fragments were gel-purified using Wizard SV Gel and PCR Clean-Up System (Promega Corp., WI, USA). For methyl-DNA bead conjugation (Fig. 7c–e), a portion of the DNA amplimer was methylated using the CpG methyltransferase, M.SssI (New England BioLabs, MA, USA) according to the recommendations of the manufacturer. Methylation reaction products were purified using Wizard SV Gel and PCR Clean-Up System (Promega) and the reaction efficiency was assessed by gel electrophoresis following challenge with the methylation-sensitive restriction enzyme, *Hpa*II (New England BioLabs). Streptavidin-coupled beads (2.8 μm diameter) were decorated with DNA using Dynabeads kilobaseBINDER Kit (Life Technologies, USA) according to the recommendations of the manufacturer. Briefly, magnetic latex microbeads from 4 μl of suspension were mixed on a rotator for 3 h at room temperature (25 °C) with 3 μg of biotinylated DNA fragment in binding buffer in 40 μl of binding reaction. The beads were harvested magnetically, washed, resuspended in nuclease-free water and stored in aliquots at -20 °C until required. Immediately prior to injection, beads were mixed with 3% (w/v) PVP (average *M*_r_, 360,000) in M2 medium. Microinjection was performed as for ICSI^56^, coinjecting 5–7 beads per oocyte or embryo instead of sperm heads.

To validate the method in our system, we injected DNA-beads into mII oocytes and evaluated microtubule formation and histone accumulation by DNA staining and immunofluorescence microscopy (Supplementary Fig. 12a–c). In all experiments, we showed that DNA-beads injected into mII oocytes were stained with Hoechst 33342 or propidium iodide, in a DNA-dependent manner (Supplementary Fig. 12a). Beads in mII oocytes stained with an antibody against acetylated H4K12ac in a DNA-dependent manner that was independent of DNA methylation (*n*≥10; Supplementary Fig. 12b). As previously reported^32^, DNA-beads induced microtubule nucleation regardless of 5mC status (*n*≥11), whereas beads lacking DNA failed to do so (*n*=11) (Supplementary Fig. 12c). The kinetics of cRNA-encoded histone H3.3 deposition were similar for DNA-beads and ICSI controls (Supplementary Fig. 10).

### Statistical analysis

Experiments were performed on ≥2 days. The number of samples (*n*) per experiment reflects power calculation and oocyte and embryo survival after manipulation. All samples were randomly collected; that is, we did not knowingly select different classes of healthy oocytes or embryos except where stated, and no data are selectively excluded. Data analysis was performed with or without blinding. Statistical differences between pairs of data sets were analysed by a two-tailed unpaired *t*-test. One-way ANOVA followed by a Tukey–Kramer *post hoc* test was used for multiple comparisons. The log-rank test was used to compare mouse longevity plots created by the Kaplan–Meier method. Values of *P*<0.05 were considered statistically significant. For model-assessment of possible ICSI-phenotype contribution to developed embryos, we assumed that two embryonic phenotypes I and II were characterized by respectively an elevation or diminution of certain modifications (for example, 5mC and 5hmC). The estimated probability for development to term is *q*_I_=0.103 for phenotype-I (according to 24/232 for phICSI-derived embryos) and *q*_II_=0.451 for phenotype-II (according to 107/237 for ICSI-derived embryos) (Supplementary Table 1). A nominal phenotype-I population may contain an unknown phenotype-II fraction, *ρ*. Subsequently, the model combines (1) the probability for observing phenotype-I associated histone states in *k* out of *k* experiments in the mixed population, and (2) the cumulative probability for the event that at least *n*=1,2,... out of *N*=232 individuals developing to term derive from phenotype-II.

Ad (1) The probability of observing *k* histone-unmodified cases (phenotype-I) in *k* experiments is binomial *p*(*k*|*k*, 1-*ρ*) distributed.

Ad (2) The probability of observing m phenotype-II and (*N*—*m*) phenotype-I cases in *N* experiments is binomial *p*(*m*|*N*, *ρ*). The probability that respectively *n* and (*N*—*n*) cases develop to term is the product of binomial *p*(*n*|*m*, *q*_II_) and *p*(*N*—*n*|*N*—*m*, *q*_I_). Marginalization with respect to m


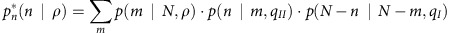


results in the probability for the combined two-step process


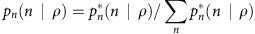


Finally, the cumulative probability for observing at least *n* individuals is


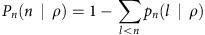


Taking (1) and (2) as independent evidence and assuming *ρ* to be the same in both types of experiments, the overall probability is the product


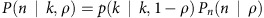


Supplementary Figure 13 shows *P* as a function of *ρ* for different values of *n* and *k*=20 (5hmC). Accordingly, the production of 9 or more phenotype-II-derived embryos out of a total of 24 can be excluded on the 5% significance level, that is, *P*(9)≤0.0465, while the probability that all embryos derive from phenotype-II is *P*(24)≤9.84e−17. For *k*=9 (5mC), the same is true for 14 or more embryos, that is, *P*(14)≤0.0493, and for all embryos *P*(24)≤1.05e−16. Regarding both evidences for *k*=9 and *k*=20 as independent and multiplying both binomials results in significance starting from *n*=7, that is, *P*(7)≤0.0465, and *P*(24)≤9.32e−17. The simplifying assumption of only two phenotypes limits the model applicability to either only one phenotype-determining modification or (equivalently) multiple but 100%-correlated modifications. Allowing for two independent modifications A and B, would require a more involved four-phenotype-model. However, our data suggest high correlation of A and B.

### Single-cell whole transcriptome amplification

ICSI and phICSI 2-cell embryos were produced by injecting B6D2F1 sperm respectively into B6D2F1 oocytes or parthenogenotes. For phICSI-13, blastomeres were separated from 2+1 embryos (Fig. 1e). In all cases, a random sample of the embryos was cultured to confirm preimplantation developmental viability (Supplementary Table 1). For transcriptome amplification, embryos were exposed to acid Tyrode's solution (pH2.5; Sigma) to dissolve the zona pellucida and allowed to recover for 1 h in KSOM in humidified 5% CO_2_ (v/v air) at 37 °C. Embryos were then gently triturated in Ca^2+^-free, Mg^2+^-free M2 medium to separate the blastomeres and the extant polar body if there was one. ICSI blastomeres were collected 27 h post-ICSI and the binuclear blastomeres of phICSI 2+1 embryos 27 h after the initiation of SrCl_2_ exposure. One blastomere per tube was collected in a minimal volume into 5.4 μl of lysis buffer (Active Motif) containing 10 ng of tRNA (Roche), 1 μg of protease (Active Motif), and 1 μl of 37.5 μM biotinylated oligo-dT peptide nucleic acids (PNAs; Active Motif) and flash-frozen in liquid N_2_ until extraction, reverse transcription and global amplification of blastomere mRNA^36^,^65^. Proteolytic digestion was performed by incubating samples for 10 min at 45 °C, followed by inactivation of protease at 75 °C for 1 min, and annealing of PNA to poly(A) tails of mRNAs, at 22 °C for 15 min. PNA-mRNA complexes were precipitated in magnetic force field using streptavidin-conjugated metal beads. While precipitating, bead pellets were washed with 10 μl of wash buffer 1 (50 mM Tris-HCl, 75 mM KCl, 10 mM DTT, 0.25% [v/v] Igepal), 20 μl of wash buffer 2 (50 mM Tris-HCl, 75 mM KCl, 10 mM DTT, 0.5% [v/v] Tween-20), and again with 20 μl of wash buffer 1 (supernatant was removed after each washing step). Solid phase reverse transcription was performed for 45 min under rotation at 44 °C, in a 20 μl reaction containing 0.5 mM of each dNTP (GE Healthcare), 200 U of SuperScript II reverse transcriptase (Invitrogen), 0.25% (v/v) Igepal, 5 mM DTT, 30 μM of C_15_GTCTAGAN_8_ primer, 15 μM of C_15_GTCTAGACTTGAGT_24_VN primer (Metabion), and 1 × first strand buffer (Invitrogen). Primers were annealed at room temperature for 10 min, before addition of the enzyme. Following reverse transcription, beads were precipitated in magnetic racks and washed in 20 μl of wash buffer 3 (50 mM KH_2_PO_4_, 1 mM DTT, 0.25% (v/v) Igepal) and resuspended in 10 μl of buffer for tailing (4 mM MgCl_2_, 0.1 mM DTT, 0.2 mM dGTP, 10 mM KH_2_PO_4_). Reaction mixtures were overlaid with 40 μl of mineral oil, and the cDNA single strands released from beads by heating to 95 °C for 5 min followed by incubation on ice for 3 min. Addition of dGTPs on 5′ termini of single stranded cDNAs was performed by adding 10 U of terminal dNTP transferase (TdT; USB-Affymetrix) and incubating the mixture for 60 min at 37 °C. After inactivation of TdT at 70 °C for 5 min, we added 35 μl of whole transcriptome amplification (WTA) reaction mix 1 (4 μl of buffer I (Expand Long Template, Roche) and 3% (v/v) deionized formamide). Hotstart PCR was performed by incubating the sample at 78 °C and adding 5.5 μl of WTA reaction mix 2 (3.2 mM each dNTP, 12 mM TCAGAATTCATGC_15_ primer, and 7.5 U of PolMix (Expand Long Template, Roche)). WTA consisted of 40 cycles in an MJ Research PCR cycler: 20 cycles of 15 s at 94 °C, 30 s at 65 °C, and 2 min at 68 °C followed by 20 cycles with an increase of elongation step of 10 s per cycle, followed by a final cycle with 7 min of elongation.

For quality control^36^,^65^, 0.5 μl of each WTA product was used as a template for end-point PCR to amplify each of the three transcripts: β2-microglobulin, β-actin and GAPDH. The primers used were: β-actin forward, CAGCTTCTTTGCAGCTCCTT); β-actin reverse, CTCGTCACCCACATAGGAGTC; β2-microglobulin forward, TGGTGCTTGTCTCACTGACC; β2-microglobulin reverse, CCGTTCTTCAGCATTTGGAT; GAPDH forward, GAAGGGCATCTTGGGCTAC; GAPDH reverse, GCCTCTCTTGCTCAGTGTCC. PCR reactions consisted of 1 × PCR buffer (PAN-Biotech GmbH, Germany), 0.4 μM each primer (Eurofins), 5 μg BSA (Roche), 0.5 U Taq polymerase (PAN-Biotech), and 0.1 mM each dNTP (GE Healthcare). PCR products were visualized on 1.5% (w/v) agarose gels stained with ethidium bromide. Only samples that were positive for all three transcripts were used for microarray analysis^36^,^65^.

### Sample labelling and microarray hybridization

Labelling of primary whole transcriptome amplified (WTA) product was by PCR with Cy5-labelled primers. Reaction mix contained 5 μl of buffer I (Expand Long Template, Roche), 3% (v/v) deionized formamide, 0.35 mM each dNTP, 2.5 μM 5'-U*CAGAAU*TCAU*CCC*CCCC*CCCC*CCCC*-3' primer (*denotes nucleotides conjugated with Cy5 fluorophore; Metabion), 7.5 U of PolMix (Expand Long Template, Roche) and 1 μl of primary WTA product in a final volume of 49 μl. PCR parameters were: one cycle with 1 min at 95 °C, 11 cycles with 15 s at 95 °C, 1 min at 60 °C, and 3 min 30 s at 65 °C, 3 cycles where the elongation time was increased 10 s per cycle, and finally one cycle with an elongation time of 7 min. Labelled products were purified using a PCR purification kit (Qiagen) according to the instructions of the vendor. Purified Cy5-labelled DNA was denatured by incubation for 5 min at 95 °C followed by incubation on ice. Hybridization solution was prepared by mixing 42 μl of denatured Cy5-labelled DNA, 55 μl of 2x HiRPM hybridization buffer (Agilent), 11 μl of 10 × GE Blocking agent (Agilent), 4 μl of 25% (v/v) Tween-20, and 4 μl of 25% (v/v) Igepal. Four 100 μl samples of hybridization mix were overlaid on 4 hybridization fields of Agilent Whole Mouse Genome (4x44K) Oligo Microarray with SurePrint (G4122F) microarray slides and incubated for 17 h at 65 °C under constant rotation. After hybridization, slides were washed in Agilent Wash buffer 1 for 1 min on a shaker, in darkness, and incubation continued in Agilent Wash buffer 2 pre-warmed to 37 °C. Slides were dried by washing for 30 s in acetonitrile and scanned on a GenePix 4400 A scanner (Molecular Devices). Numerical readouts of fluorescence intensities (GPR files) were generated using GenePixPro 7 (Molecular Devices).

### Bioinformatics

The quality of whole transcriptome amplification was assessed by control PCR. Gene expression data were quality assessed by inspection of chip raw images and gene expression frequency distributions. All experiments were of sufficiently high quality for further bioinformatic analysis (16 and 8 expression profiles for ICSI and phICSI, respectively). Raw gene expression data were background corrected (limma R-package, normexp method)^66^,^67^, log_2_-transformed and normalized by quantile normalization. Technically replicated probes (identical Agilent IDs) were replaced by their median per sample. Gene ranking was performed according to moderated *t*-statistics (limma R-package). For graphical display and functional annotation probes targeting the same gene were disambiguated by retaining only the probe with the lowest *P*-value. The normalized heatmap (Fig. 8c) employed Euclidean distance and complete linkage for agglomerative clustering. Enrichment for biological process annotation associated with genes most differentially expressed between ICSI and phICSI was obtained by querying the DAVID database^68^ for GO BP/5, GO BP/FAT and KEGG, Reactome, BBID, BioCarta, and Panther pathway annotation using the R-package RDAVIDWebService^69^ for submitting stepwise prolonged gene-lists starting from the top 50, 100, 150, 200, 250 and so on. top-ranking genes up to a length of 550 corresponding to an FDR-adjusted *P*-value of 0.15. If identical annotation terms were returned for different lists only the one with the lowest EASE *P*-value was retained. For assessing randomness of gene ranking in the ICSI_1 versus ICSI_2 comparison, all 6,435 possible 8+8 array combinations were enumerated and *P*-values calculated (limma R-package). The resulting curves were kernel smoothed in two-dimensional and graphically displayed as density. From the 128 possible combinations respecting the experimental 2-cell pairing, the one with the highest number of FDR-adjusted *P*-values <0.05 (corresponding to the largest difference between ICSI_1 and ICSI_2) was selected for graphical display. In the ICSI versus phICSI comparison, 10,000 of the 735,471 possible 16+8 array combinations were analogously calculated to obtain the corresponding graphical display. Array data are deposited at the Gene Expression Omnibus (GEO) with the accession number GSE60595.

### Ratiometric PCR (qPCR)

Relative transcript levels were quantified by transferring single embryos (1 per tube) in a minimal volume (<0.5 μl) of 0.1% (w/v) Sarkosyl (Teknova, Hollister, CA, USA) containing 10 ng μl^−1^ tRNA (Hoffmann-La Roche, Ch), heated at 65 °C for 5 min and used to programme cDNA synthesis primed with oligo(dT)_20_ and random 8-mers (each at 30 mM) in a 21 μl reaction volume containing 200 U SuperScript III reverse transcriptase (Invitrogen Corp., CA, USA). Real-time qPCR was in an ABI 7500 Real Time PCR System (Applied Biosystems, CA) in reactions (20 μl total) containing 1–2 μl of the template cDNA, forward and reverse primers (Supplementary Table 2; each at 5 nM) and 12.5 μl of Power SYBR (ABI), using the parameters: 10 min at 95 °C, followed by 45 cycles of (15 s at 95 °C, 1 min at 58–60 °C and 35 s at 72 °C). Each sample was assayed in triplicate and given sample sets collected on at least two days. Primer sets were non-dimerizing under the conditions employed and steady state levels of transcripts were normalized against internal controls^36^.

### Data availability

Microarray data have been deposited in the Gene Expression Omnibus (GEO) database under accession code GSE60595. The authors declare that the data supporting the findings of this study are available within the article and its Supplementary Information Files or from the corresponding authors upon reasonable request.

## Additional information

**How to cite this article:** Suzuki, T. *et al*. Mice produced by mitotic reprogramming of sperm injected into haploid parthenogenotes. *Nat. Commun.* 7:12676 doi: 10.1038/ncomms12676 (2016).

## Supplementary Material

Supplementary Information Supplementary Figures 1-13, Supplementary Tables 1-2
