# Supplementary material for: Mice produced by mitotic reprogramming of sperm injected into haploid parthenogenotes
Source: Nat Commun. 2016 Sep 13;7:12676. doi: 10.1038/ncomms12676 (PMC5027272; doi:10.1038/ncomms12676)
Supplement: Supplementary Information — Supplementary Figures 1-13, Supplementary Tables 1-2 [file ncomms12676-s1.pdf]

Supplementary tables and figure legends

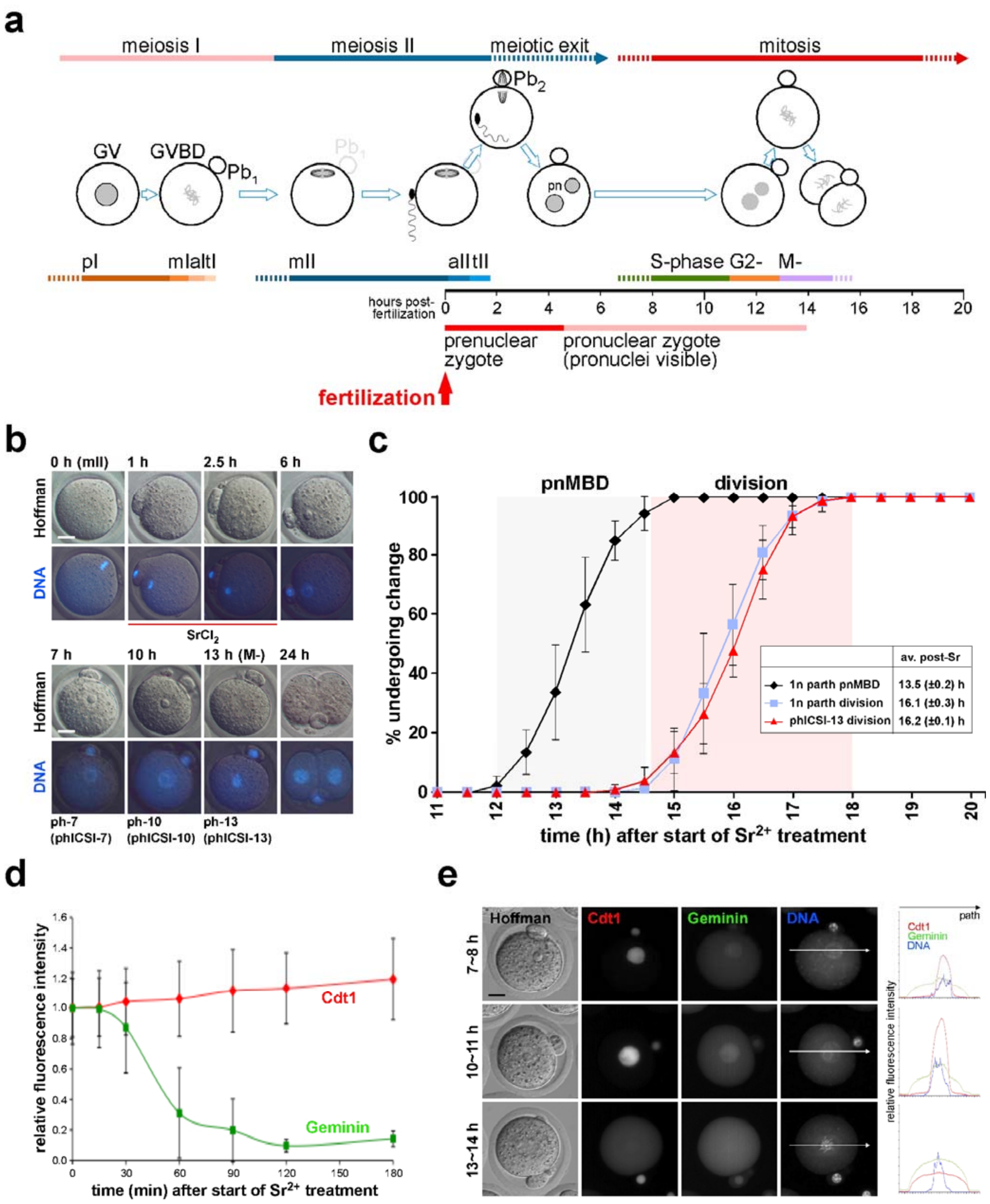

Supplementary Figure 1 Characteristics of mouse preimplantation development relevant to phICSI. (**a**) Schematic representation showing the transition from meiosis to a mitotic embryo following fertilization. GV, germinal vesicle (immature oocyte nucleus); GVBD, GV breakdown; Pb_1, first polar body; Pb_2, second polar body; pI, mI, aI and tI respectively refer to the first meiotic prophase, metaphase, anaphase and telophase, with corresponding nomenclature for the second meiosis. (**b**) Paired Hoffman modulation (upper) and DNA labelling (Hoechst 33342) images of oocytes or haploid parthenogenetic embryos at the times shown (h) after initial exposure to 10 mM $SrCl_2$. mII, metaphase II; M, mitotic. (**c**) Timing of 1- to 2-cell division in phICSI and parthenogenesis. Haploid parthenogenotes (1n parth) undergo pronuclear membrane breakdown (pnMBD; *n*=140) followed by division to 2-cells (*n*=140) at the times indicated (± s.d. from 2 experimental days). The average timing of this division after the start of Sr^{2+} treatment (av. post-Sr) is not significantly different (*n*=250) in phICSI 13 h after Sr^{2+} treatment (phICSI-13) and parthenogenotes. (**d**) Quantification (± s.d. from 2 experimental days) of cRNA-encoded Geminin-Venus (Gemn) and Cdt1-mCherry (Cdt1) fluorescence levels at the times (min) after initial exposure to 10 mM $SrCl_2$ (*n*=25). (**e**) Grouped Hoffman modulation (leftmost) and fluorescence images showing Geminin-Venus (Geminin) and Cdt1-mCherry (Cdt1) levels at the times indicated after initial Sr^{2+} exposure. Representative fluorescence plots are shown for paths traversing chromatin. Scale bars in (**b**) and (**e**), 20 μm.

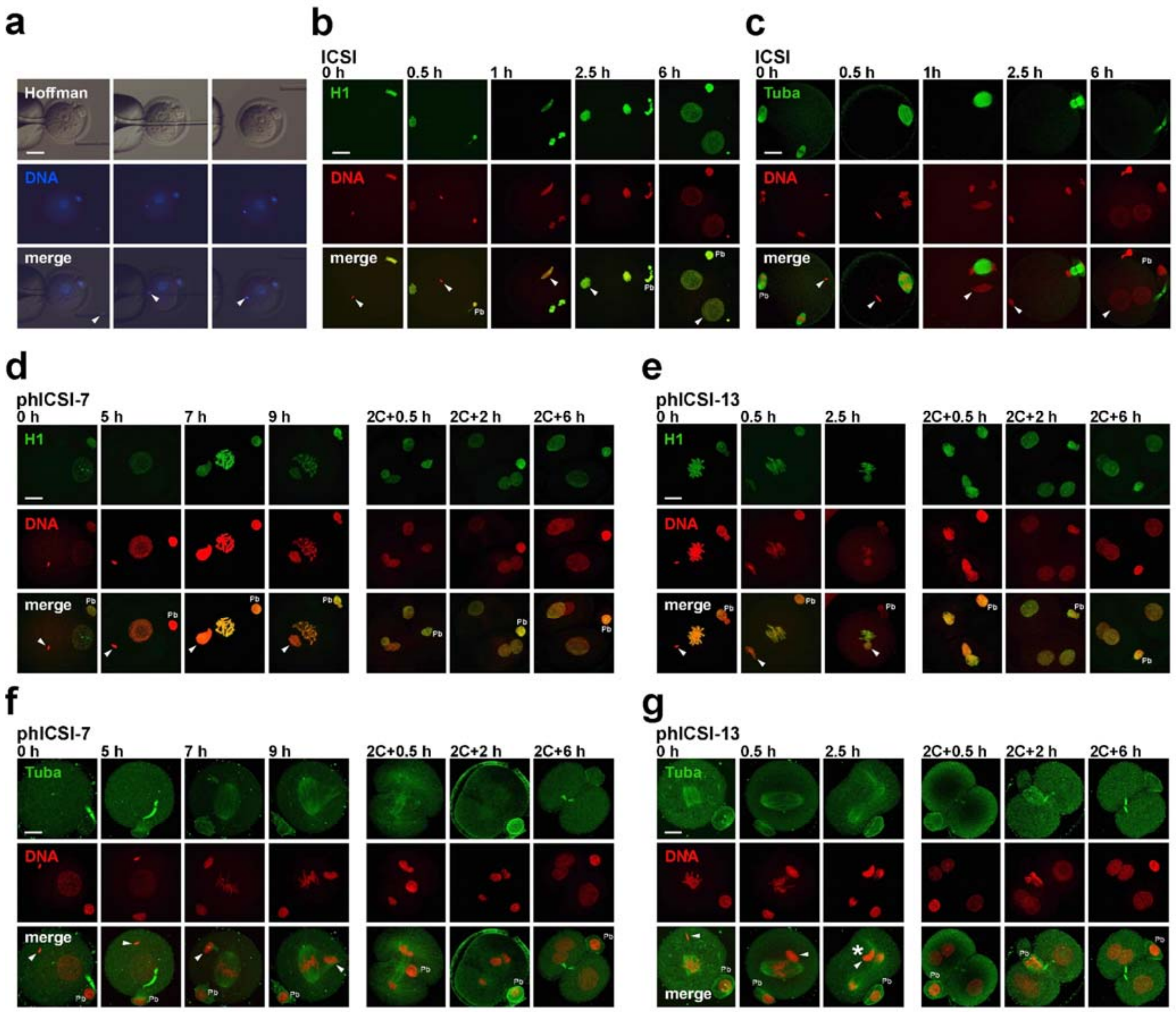

Supplementary Figure 2 Intracellular dynamics in phICSI. (**a**) Injection of sperm heads (arrowheads) as per Figure 1a, but into parthenogenotes 7 h after the start of $SrCl_2$ treatment (phICSI-7); images for phICSI-10 appear similar. (**b**) Representative confocal images (grouped vertically) showing propidium iodide (PI) staining (DNA) or linker histone H1 (H1) immunofluorescence at the times shown (h) after sperm injection (ICSI) into mII oocytes (*n*=4 per time point). (**c**) Staining as for (**b**), but with tubulin-α (Tuba) immunofluorescence (*n*=5 per time point). (**d**) Vertically-grouped fluorescence images for PI or H1 immunostaining at the times shown after the start of Sr^{2+} treatment or after 1-cell to 2-

cell division (2C+0.5 h, *etc.*) in phICSI-7 (*n*=4 per time point). (**e**) Staining as for (**d**), but in phICSI-13 (*n*=4 per time point). (**f**) Counterpart (*n*=10 per time point) of (**d**), except that immunofluorescence shows Tubulin-α (Tuba). (**g**) Counterpart (*n*=13 per time point) of (**e**), except that immunofluorescence shows Tubulin-α (Tuba), indicating incomplete microtubule assembly (asterisk). Scale bars, 20 μm.

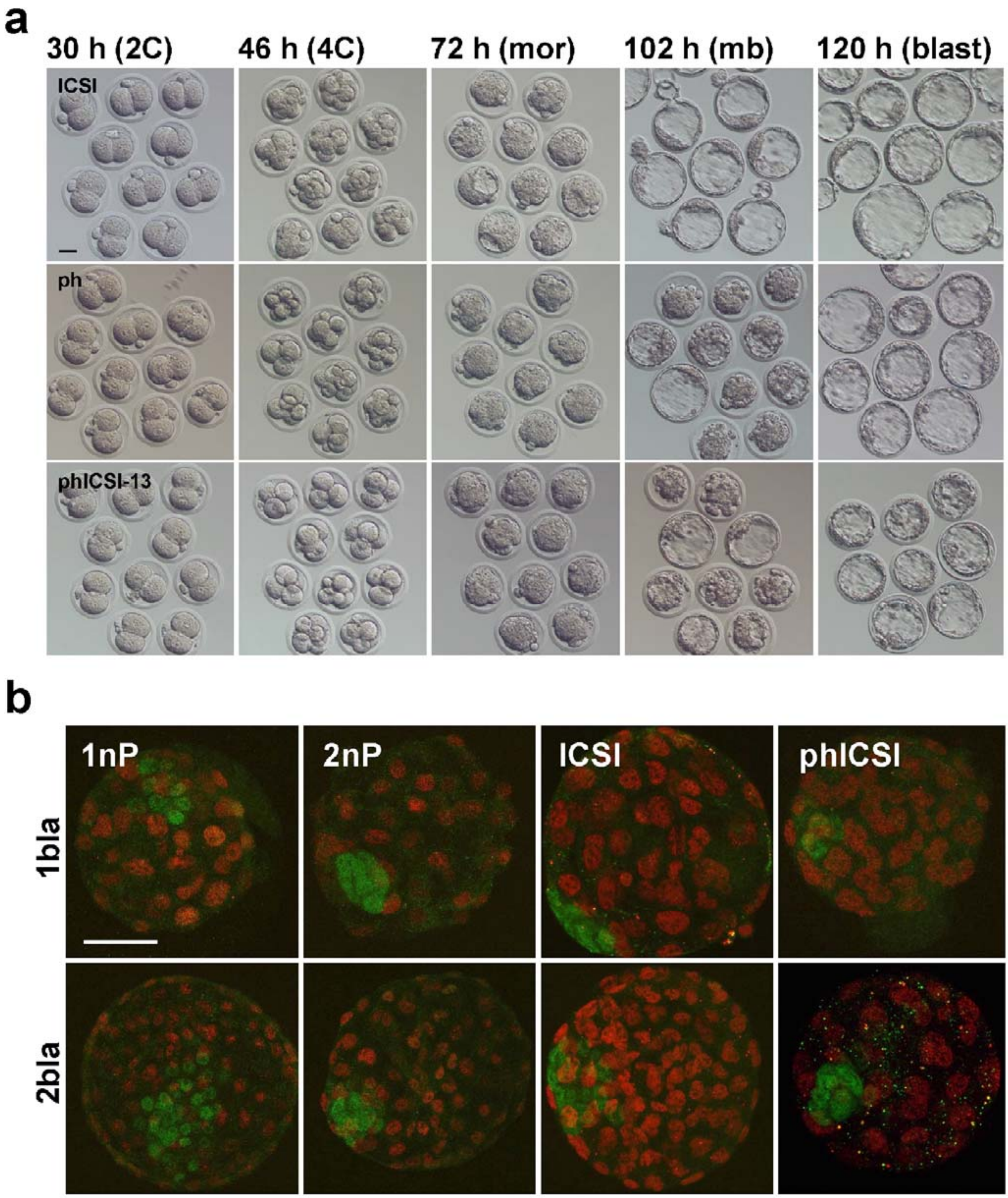

Supplementary Figure 3 Preimplantation development following phICSI. (**a**) Hoffman images of different stages of *in vitro* preimplantation development for the embryo types indicated for each row. Embryos are: control sperm injection (ICSI); 1n parthenogenote (ph)

and phICSI at 13 h (phICSI-13). Embryo stages are: 2-cell [30 h (2C)], 4-cell [46 h (4C)], morula [72 h (mor)], morula-blastocyst [102 h (mb)] and blastocyst [120 h (blast)]. (**b**) Fluorescence images showing Oct4 (green) and/or Cdx2 (red) immunopositive cells in E5.0 blastocysts. 1bla, blastocysts derived from 2-cell embryos in which one blastomere was ablated; 2bla, blastocysts derived from 2-cell embryos without blastomere ablation; 1nP, haploid parthenogenote; 2nP, diploid parthenogenote. Scale bars, 50 μm.

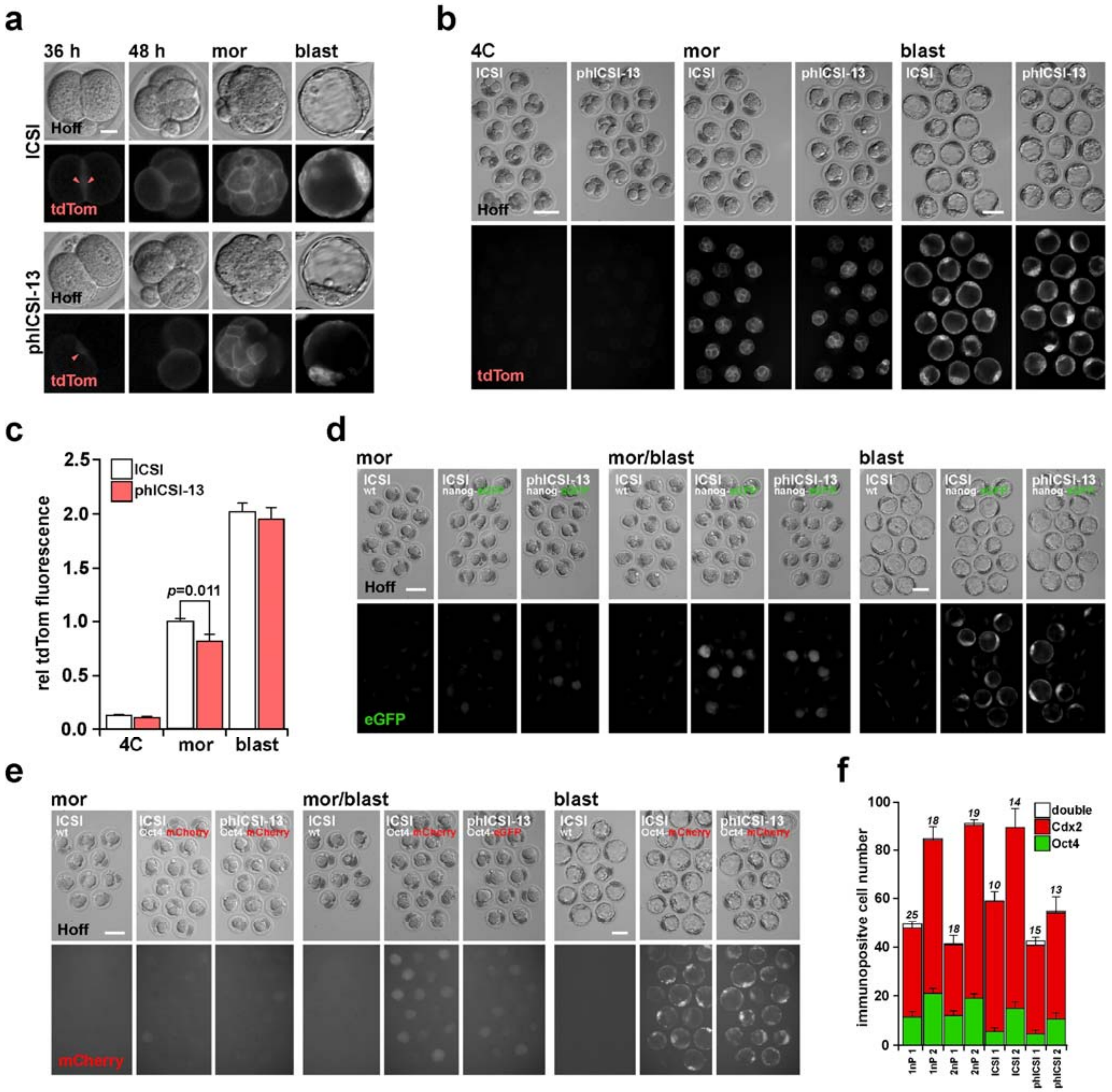

Supplementary Figure 4 Development and expression of sperm-borne transgenes and pluripotency markers in phICSI-13. (**a**) Vertically paired Hoffman (upper) and fluorescence micrographs of embryos generated by injecting sperm carrying a ubiquitously-expressed membrane *tdTomato* (tdTom) transgene into mII oocytes (ICSI) or parthenogenotes (phICSI-13); tdTom expression (arrowheads) was detected 36 h after meiotic resumption. Parthenogenetic blastomeres in phICSI-13 lack *tdTomato*. (**b**)

Micrographs of embryos derived as for (**a**), except that one blastomere was destroyed at the 2-cell stage, producing -1bla embryos. In phICSI-13-1bla the presumptive uniparental blastomere was destroyed. (**c**) Histogram showing relative intensities of tdTom expression of (**b**) in ICSI-1bla (open) and phICSI-13-1bla (red) embryos (± s.e.m.; n=30 for each set from 2 experimental days). Differences below the 5% confidence limit ($p<0.05$; unpaired *t*-test) are shown. (**d**) Vertically paired Hoffman (upper) and fluorescence micrographs of embryos generated as per the -1bla embryos of (**b**), by injecting mII oocytes (ICSI) or parthenogenotes (phICSI-13) with sperm carrying *eGFP* knocked into the *Nanog* locus (pNanog-eGFP heterozygotes), or with control ICSI using wild-type sperm. (**e**) Vertically paired Hoffman (upper) and fluorescence micrographs of embryos generated as per (**d**) by injecting mII oocytes (ICSI) or parthenogenotes (phICSI-13) with sperm carrying a transgene encoding mCherry expression driven by the *Oct4* promoter (*pOct4-mCherry*), or with control ICSI using wild-type sperm. Blastocyst images are also presented in Figure 1c and are included for completeness. (**f**) Histogram showing average numbers (*n* above each column, ± s.e.m. from 2 experimental days) of Oct4 (green) and/or Cdx2 (red) immunopositive cells in E5.0 blastocysts. 1, blastocysts derived from 2-cell embryos in which one blastomere was ablated; 2, blastocysts derived from 2-cell embryos without blastomere ablation; 1nP, haploid parthenogenote; 2nP, diploid parthenogenote. Scale bars in (**a**), 20 μm and in (**b**), (**d**) and (**e**), 100 μm.

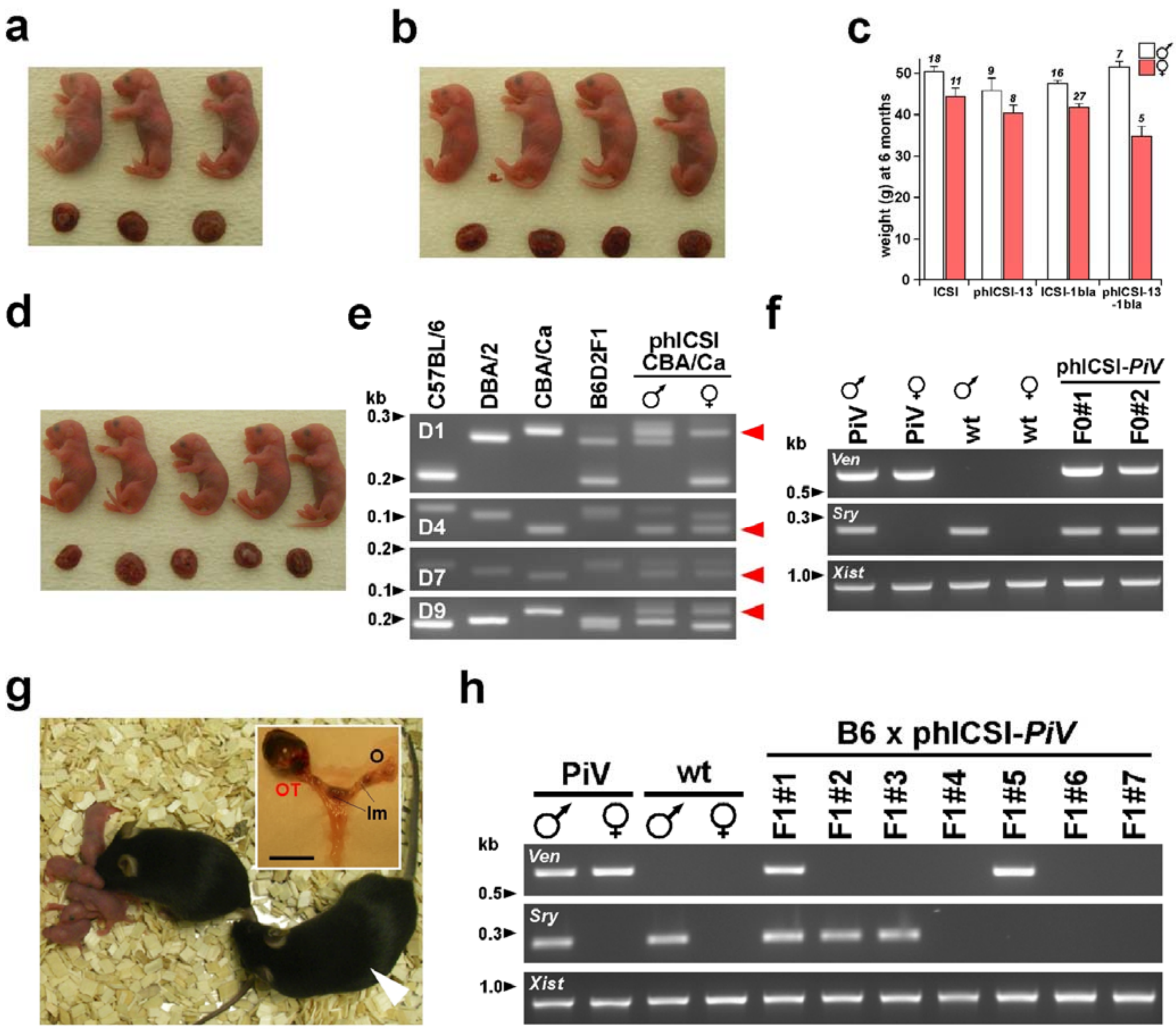

Supplementary Figure 5 phICSI supports term development in different strains. (**a**) Images of neonates and their associated placentae from phICSI-7 embryos generated with B6D2F1 (hybrid) sperm. (**b**) Images as per (**a**), except for phICSI-10 embryos and placentae. (**c**) Body weights (± s.e.m.) of ICSI- and phICSI-13-derived offspring at 6 months; *n* is indicated above each column. (**d**) Images of neonates and their associated placentae from phICSI-13-1bla generated by injecting sperm from the inbred strain, CBA/Ca. (**e**) PCR analysis of four microsatellite markers shows that CBA/Ca-specific variants (red arrowheads) are present in genomic DNA from female and male B6D2F1 x CBA/Ca phICSI-

13-1bla offspring. (**f**) Genomic PCR of the *PiV* transgene (*V*) and X (*Xist*) and Y (*Sry*) sex chromosomes in control and phICSI-*PiV* (F0#1 and F0#2) offspring. (**g**) phICSI-*PiV* phenotypes, showing phICSI-*PiV* F0#1 (arrowhead) male with litter produced by crossing with a C57BL/6 (B6) female and the reproductive tract of B6 x phICSI-*PiV* female F1 aged 6 months (inset), with ovary (O), implantation fossae (Im) and ovarian tumour (OT), phenocopying *Plcz-ires-Venus* transgenic mice. Scale bar, 10 mm. (**h**) PCR of *PiV* transgene (*V*) and X and Y sex chromosomes in control and seven B6 x phICSI-*PiV* F1 offspring; F1#1 and F1#5 are respectively male and female *PiV* transgenic progeny.

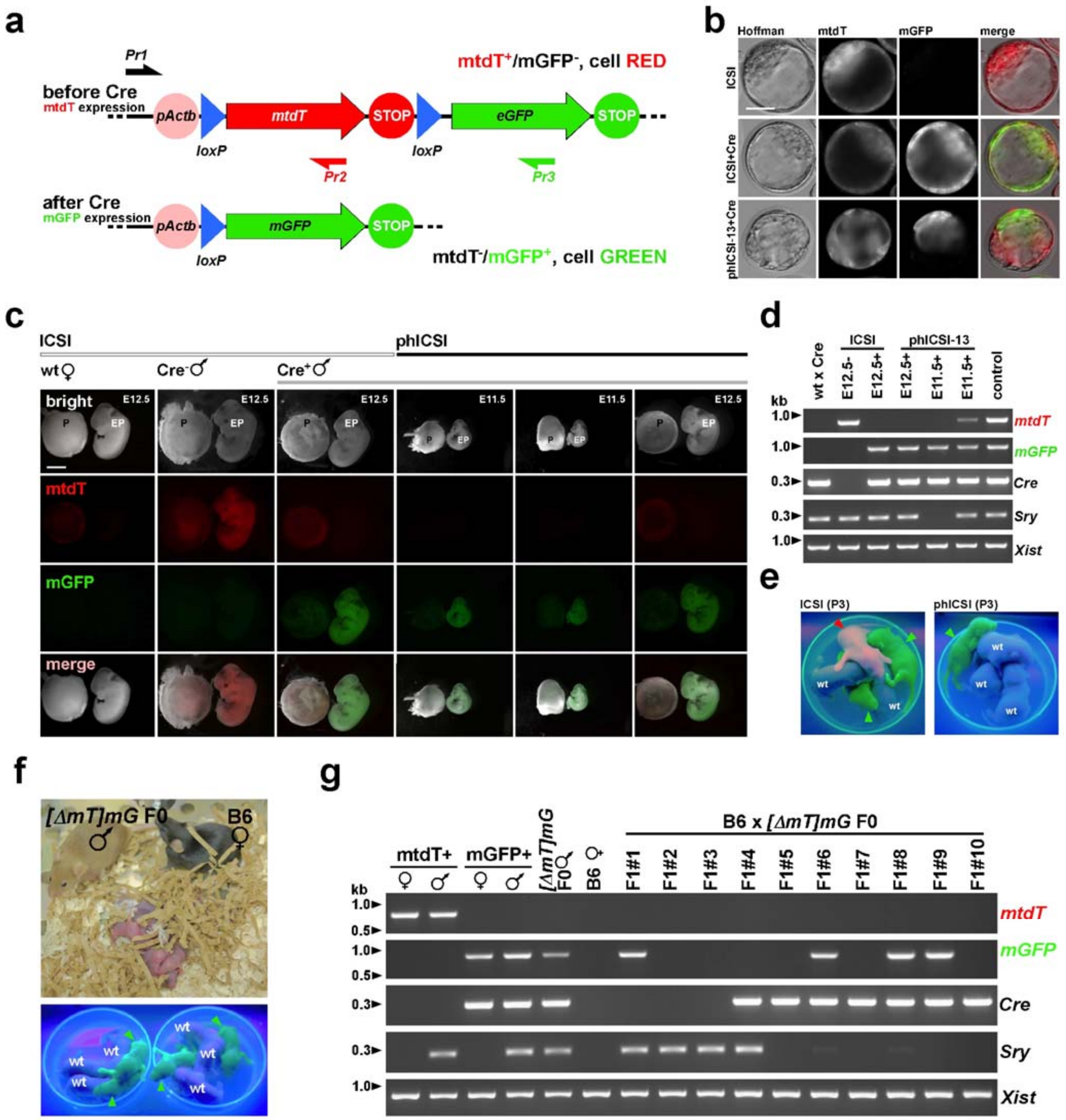

Supplementary Figure 6 Blastomere lineage tracing in phICSI. (**a**) Configuration of, and expression from the *mT/mG* locus before and after Cre-mediated excision across *loxP* sites, showing relative positions of PCR primers, Pr1-3. (**b**) Hoffman and fluorescence images following ICSI (Cre-) using oocytes from homozygous *mT/mG*$^{+/+}$ females, or ICSI (ICSI+Cre) or phICSI-13 (phICSI-13+Cre) with sperm from homozygous *pPGK-Cre*$^{+/+}$ males and

oocytes or parthenogenotes from homozygous *mT/mG*$^{+/+}$ females. Bar, 50 μm. (**c**) Bright-field (bright) and fluorescence stereomicroscopy of embryos and their associated placentae at embryonic day 11.5 (E11.5) and E12.5 after ICSI or phICSI as for (**b**). Columns show the same placenta (P) and embryo proper (EP). Controls in the leftmost and adjacent columns were derived from wild-type (wt) oocytes and *Cre* homozygous sperm (Cre$^{+/+}$). Bar, 3 mm. (**d**) PCR of genomic DNA from E11.5 and E12.5 whole embryos as indicated. Cre-expressing sperm were used except for E12.5- ICSI. mtdT, membrane tdTomato; mGFP, membrane GFP. (**e**) ICSI and phICSI offspring 3 days after delivery (P3), generated by injecting mT/mG$^{+/-}$ (red arrowhead) or mT/mG$^{+/+}$ (green arrowhead) oocytes or parthenogenotes with Cre$^{+/-}$ sperm. Active Cre excises *mTdT* to allow mGFP expression. (**f**) Germline transmission from a phICSI *[ΔmT]mG* F0 male (indicated) with an F1 litter produced by crossing with a C57BL/6 (B6) female, showing widespread green fluorescence in offspring (below; green arrowheads). (**g**) Genomic PCR of *mGFP* in controls and ten B6 x phICSI-*[ΔmT]mG* F1 offspring; *[ΔmT]mG* has been transmitted to F1#1, #6, #8, #9.

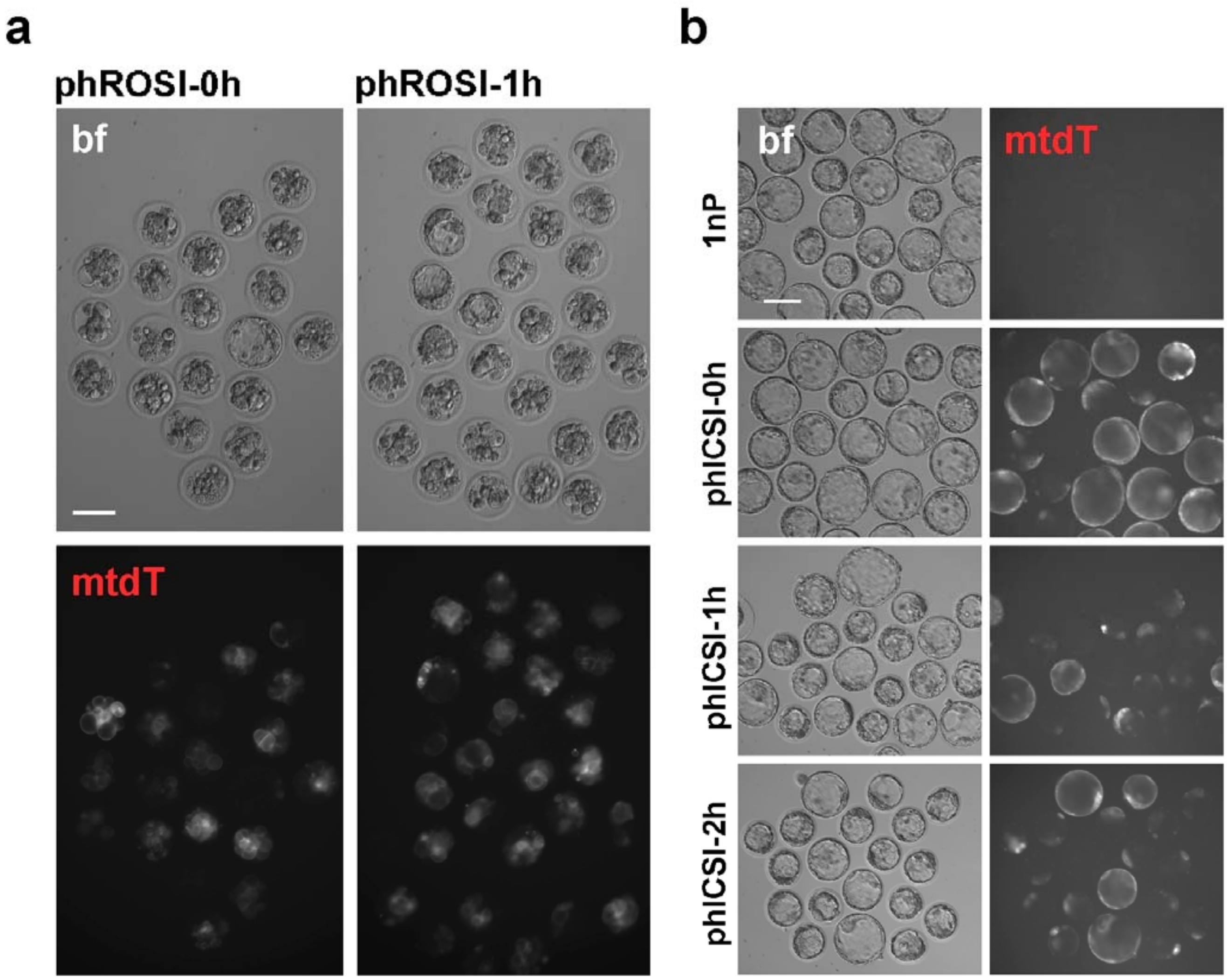

Supplementary Figure 7 Development *in vitro* following phROSI. (**a**) Vertically paired representative Hoffman modulation contrast (left, bf) and membrane tdTomato (mtdT) fluoresence images showing blastocyst development at E5.0 following ROSI into ph embryos within 30 min of pronuclear membrane breakdown (phROSI-0h) or 1 h after pronuclear membrane breakdown (phROSI-1h) as indicated. In these experiments, phROSI was from round spermatids of males homozygous for a ubiquitously-expressed *mtdT* transgene. (**b**) Images as per (**a**), but horizontally paired and showing E5.0 parthenogenetic haploid (1nP), phICSI-0h, -1h and -2h embryos. Bars, 100 μm.

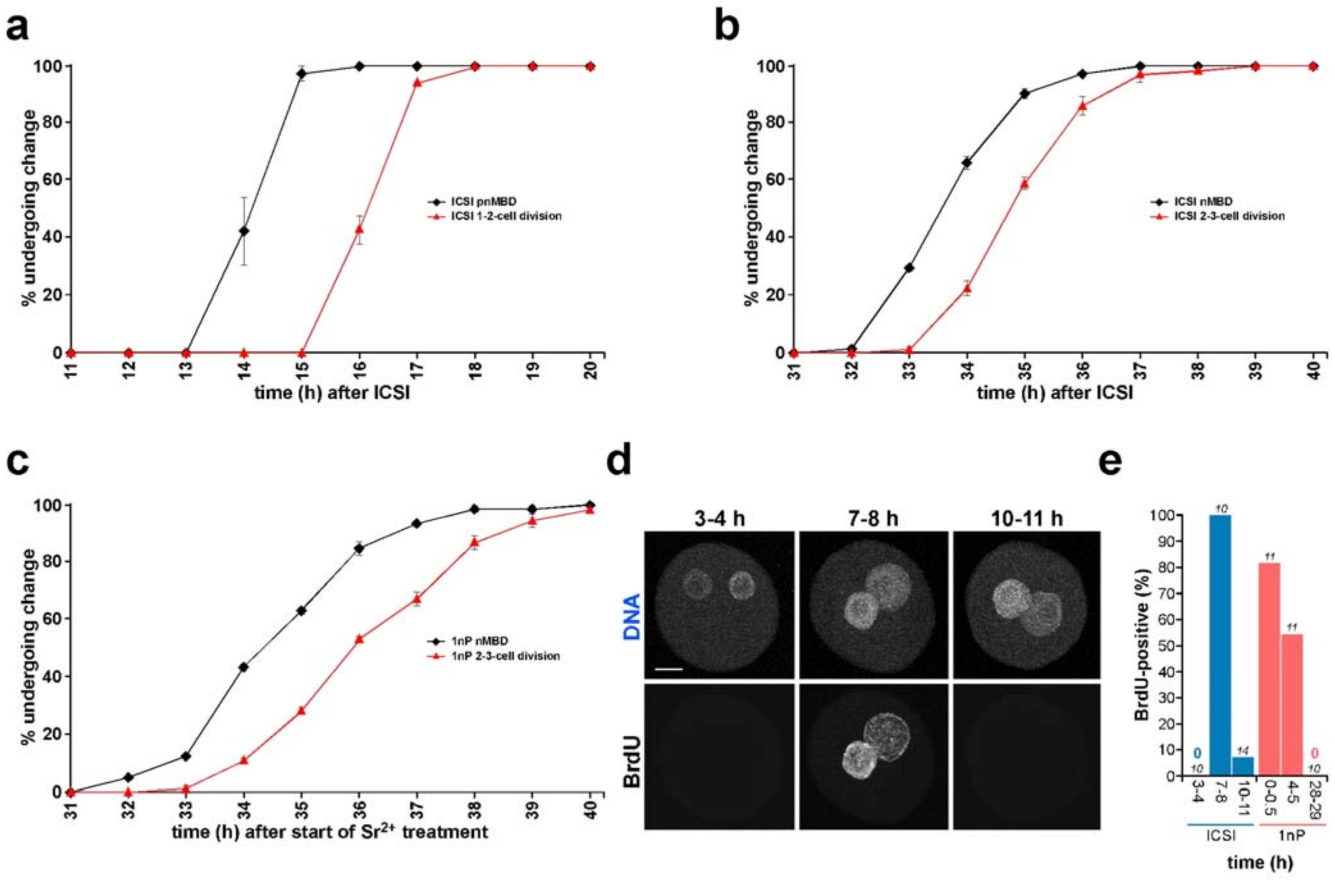

Supplementary Figure 8 Parameters for male pronuclear transfer to haploid parthenogenotes. (**a**) Timing of pronuclear membrane breakdown (pnMBD) and the first mitotic (1-cell to 2-cell) division in ICSI. (**b**) Timing of nuclear membrane breakdown (nMBD) and the second mitotic (2-cell to 3-cell) division in ICSI. (**c**) Timing of nuclear membrane breakdown (nMBD) and the second mitotic (2-cell to 3-cell) division in haploid parthenogenotes (1nP). (**d**) Fluorescence confocal micrographs indicating BrdU incorporation into newly-synthesized DNA in ICSI-derived zygotes. Zygotes were treated with 100 μM BrdU for 1 h at the times indicated after ICSI. (**e**) Percentages of BrdU-positive embryos in ICSI-derived embryos (ICSI) and parthenogenotes (1nP) treated with 100 μM BrdU for 1 h at the times indicated after ICSI, and for 1nP after cell division (0-0.5 and 4-5 h) or exposure to $SrCl_2$ (28-29 h). Staining in the 1nP 0-0.5 group was initiated 0-0.5 h after

cell division, for 1 h. All data were collected on 2 experimental days; *n* is indicated above each column. Values in (**a**) to (**c**) are ± s.e.m.

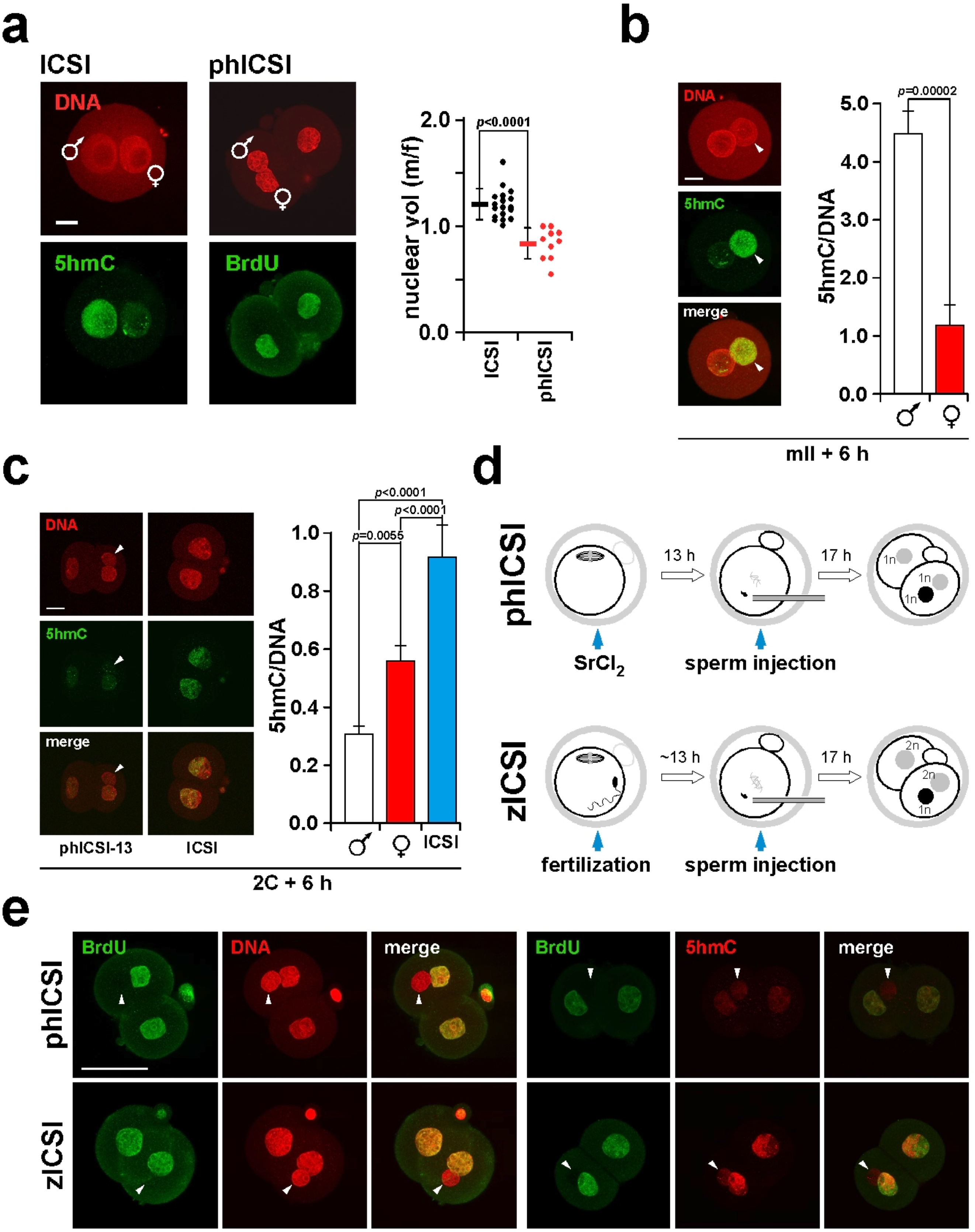
a
ICSI
phICSI
DNA
5hmC
BrdU
p<0.0001
nuclear vol (m/f)
2.0
1.0
0.0
ICSI
phICSI
b
DNA
5hmC
merge
p=0.00002
5hmC/DNA
5.0
4.0
3.0
2.0
1.0
0.0
mII + 6 h
c
DNA
5hmC
merge
phICSI-13
ICSI
p<0.0001
p<0.0001
p=0.0055
5hmC/DNA
1.0
0.8
0.6
0.4
0.2
0.0
ICSI
2C + 6 h
d
phICSI
13 h
17 h
1n
1n
1n
SrCl2
sperm injection
zICSI
~13 h
17 h
2n
2n
1n
fertilization
sperm injection
e
phICSI
zICSI
BrdU
DNA
merge
BrdU
5hmC
merge

Supplementary Figure 9 Nuclear remodeling in phICSI and zygotic ICSI, zICSI. (**a**) Vertically paired fluorescence images (left) of embryos 9 h after ICSI or 30 h after initial $SrCl_2$ exposure in phICSI-13, to show relative nuclear volumes. Staining was with propidium iodide (PI, DNA) or antibodies recognizing 5hmC or BrdU. Parental genome provenance was determined by stronger 5hmC labeling of the paternal genome in ICSI, or following culture in BrdU (to label the maternal genome) prior to sperm injection for phICSI-13. Scale bar, 20 μm. Plots show ratios of pixel areas (± s.d.) in parental nuclei following ICSI (n=18) and phICSI (n=10). Significantly different (p<0.05; unpaired t-test) pair-wise comparison is indicated. (**b**) Representative immunofluorescence images (left) of 5'-hydroxymethylcytosine (5hmC) and PI (DNA) fluorescence in a zygote 6 h post-ICSI (mII + 6 h). Arrowheads indicate paternal pronuclei. Scale bar, 20 μm. Histograms show 5hmC intensities relative to DNA in paternal and maternal chromatin (± s.e.m.). Significantly different (p<0.05; unpaired t-test) pair-wise comparison is indicated. (**c**) Representative immunofluorescence images (left) of 5hmC (green) in 2-cell embryos 6 h after division (2C + 6 h). Arrowheads indicate paternal pronuclei. Scale bar, 20 μm. Histograms (± s.e.m.) show the corresponding relative 5hmC intensities in paternal and maternal chromatin in phICSI and in the chromatin of ICSI-derived embryos (ICSI) 6 h after the first mitotic cleavage. Significant differences (p<0.05, 1-way ANOVA followed by Tukey-Kramer test) are indicated. (**d**) Schematic representation of phICSI and zygotic ICSI (zICSI), in which sperm (paternal genome shown filled in black) are injected into 1-cell embryos produced by natural mating, soon after pronuclear membrane breakdown. (**e**) Fluorescence images showing maternal DNA (BrdU, green), nuclear DNA (PI, red) or 5'-hydroxymethylcytosine (5hmC, red) 14 h after the first mitotic division (*ie* shortly before 2C→4C division) in phICSI-13 and zICSI. Arrowheads indicate paternal pronuclei. Scale bar, 50 μm. All data are from 2 experimental days.

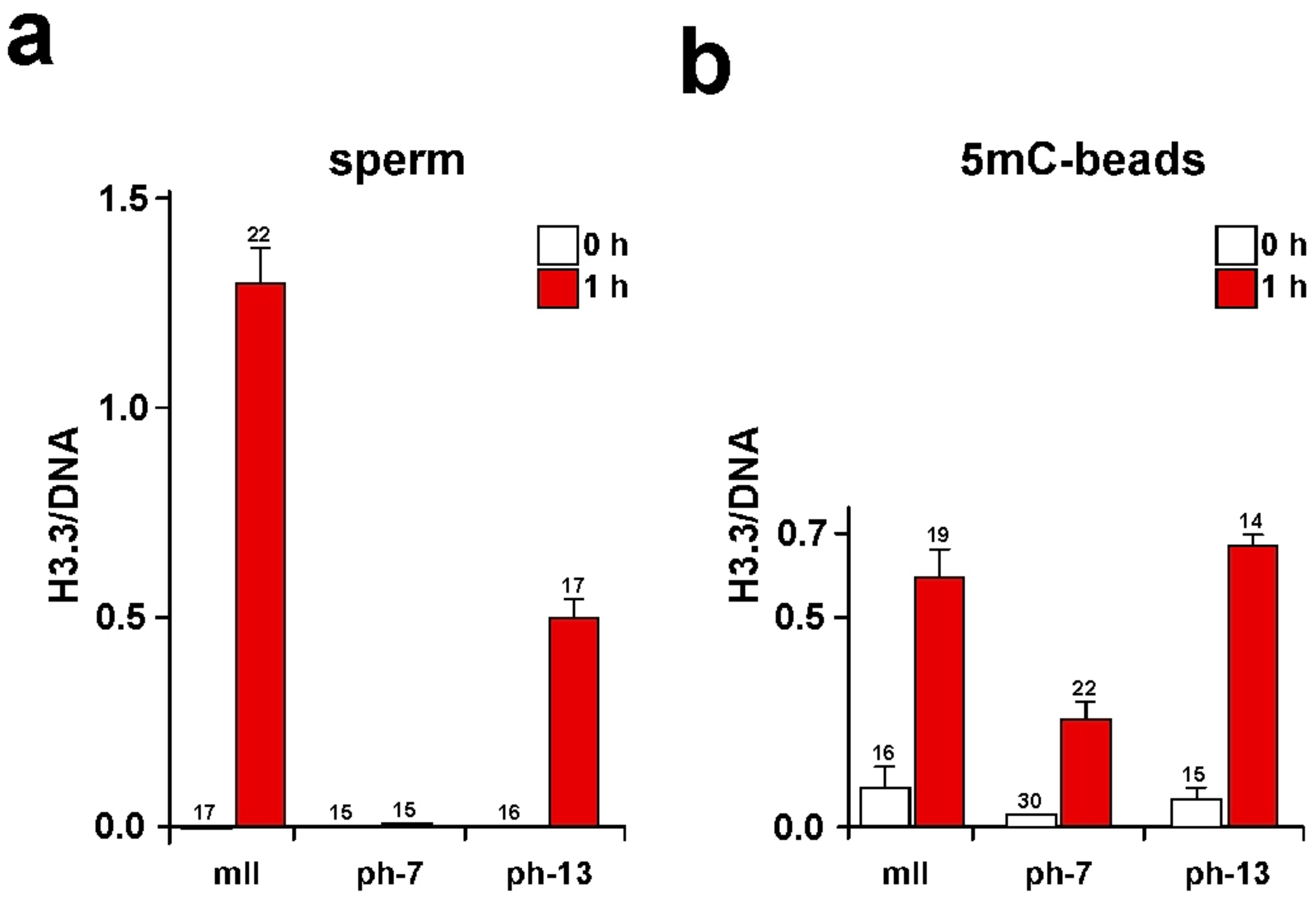

Supplementary Figure 10 Recombinant histone deposition onto paternal DNA and methyl-DNA-beads. (**a**) Histograms (± s.e.m.) indicating the acquisition of cRNA-encoded H3.3-KO2 (H3.3) by sperm heads (sperm) relative to total DNA at the times indicated (0 or 1 h) after injection. ph-7 and -13 refer to parthenogenotes 7 or 13 h after initial $SrCl_2$ exposure. (**b**) As per (**a**), except that acquisition was by 5mC-containing DNA-beads (5mC-beads). Data are from 2 experimental days; *n* is indicated above each column.

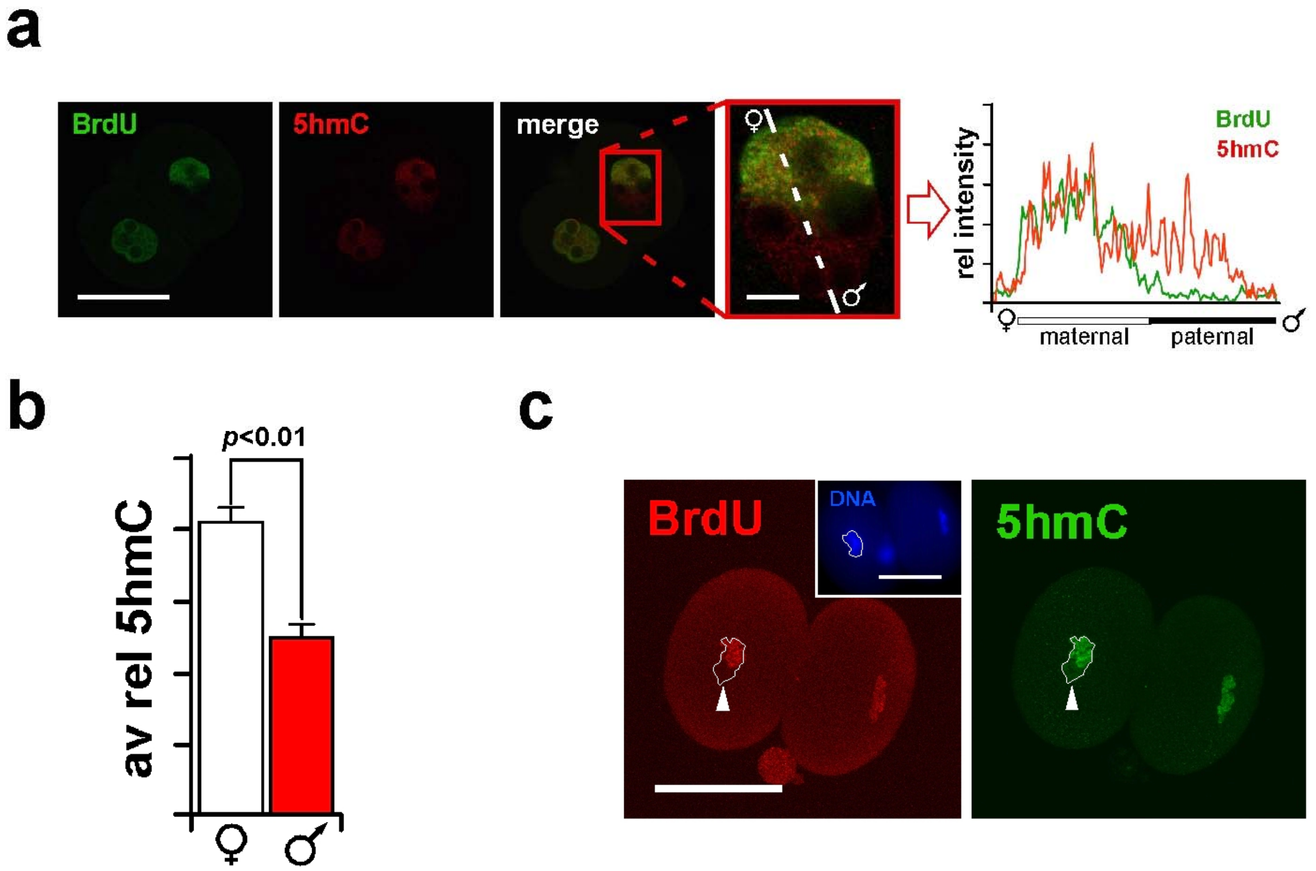

Supplementary Figure 11 DNA dynamics in 1+1 phICSI embryos. (**a**) Representative fluorescence images of maternal DNA (BrdU, green) and 5hmC (red) in a 1+1 nuclear type phICSI-13 embryo. Inset shows nucleus close-up with relative intensity plot (rightmost) along the path indicated. (**b**) Relative intensities (± s.e.m.) of the genomic 5hmC of (**a**). Significantly different (p<0.05; unpaired t-test) pair-wise comparisons are indicated. (**c**) Fluorescence confocal images of a phICSI-13 2-cell embryo, showing maternal genome (BrdU, red), 5hmC (green) and (inset) epifluorescence imaging with (Hoechst 33342, DNA). Arrowheads indicate the paternal genome. Scale bars, 50 μm and 5 μm for the inset in (**a**). All data are from 2 experimental days.

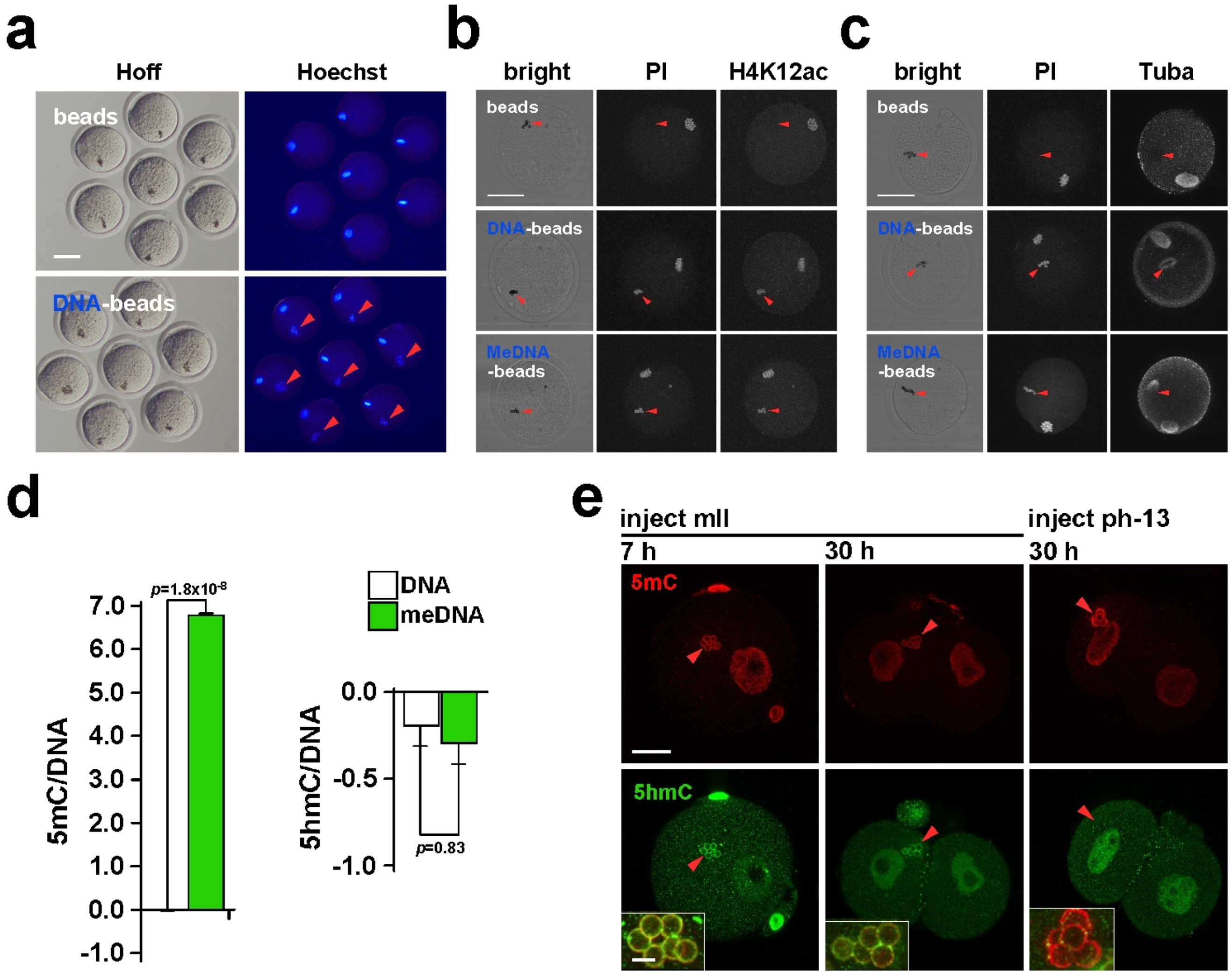

Supplementary Figure 12 DNA-bead validation. (**a**) Horizontally paired images of mII oocytes 1 h after injection with latex microbeads alone (beads) or microbeads conjugated to DNA (DNA-beads) and viewed by Hoffman modulation microscopy (Hoff) or fluorescence microscopy after DNA staining with Hoechst 33342 (Hoechst). Maternal chromosomes and DNA-conjugated beads were labeled. (**b**) Representative bright-field (bright) images of mII oocytes stained for DNA with propidium iodide (PI) or after antibody labeling of histone H4 acetylated on residue K12 (H4K12ac), 6 h after injection of microbeads alone (beads), microbeads conjugated to DNA (DNA-beads) or microbeads conjugated to DNA that had been methylated *in vitro* (MeDNA-beads). Rows represent the same oocyte. (**c**) Representative oocytes as per (**b**), except that antibody labeling was of α-Tubulin (Tuba), a

major subunit of microtubules. Scale bars in (**a**-**c**), 50 μm. (**d**) Intensities of 5mC and 5hmC in beads conjugated to control non-methylated DNA (DNA) or to 5mC-containing DNA (meDNA) (see Fig. 7c,d) relative to DNA (stained with PI) 7 h after injection into in mII oocytes ($n \geq 7$). Significantly different ($p<0.05$; unpaired *t*-test) pair-wise comparisons are indicated. (**e**) Immunofluorescence images (scale bar, 20 μm) showing 5mC (red) and 5hmC (green) on 5mC-beads in ph-7 embryos (injected at mII; $n=6$) or ph-30 embryos (injected at mII [$n=7$] or ph-13 [$n=6$]), showing merged close-ups (inset; bar, 3 μm). Beads and DNA-beads in panels (**a**-**c**) and (**e**) are indicated with arrowheads. The values of (**d**) are ± s.e.m. and all data are from 2 experimental days.

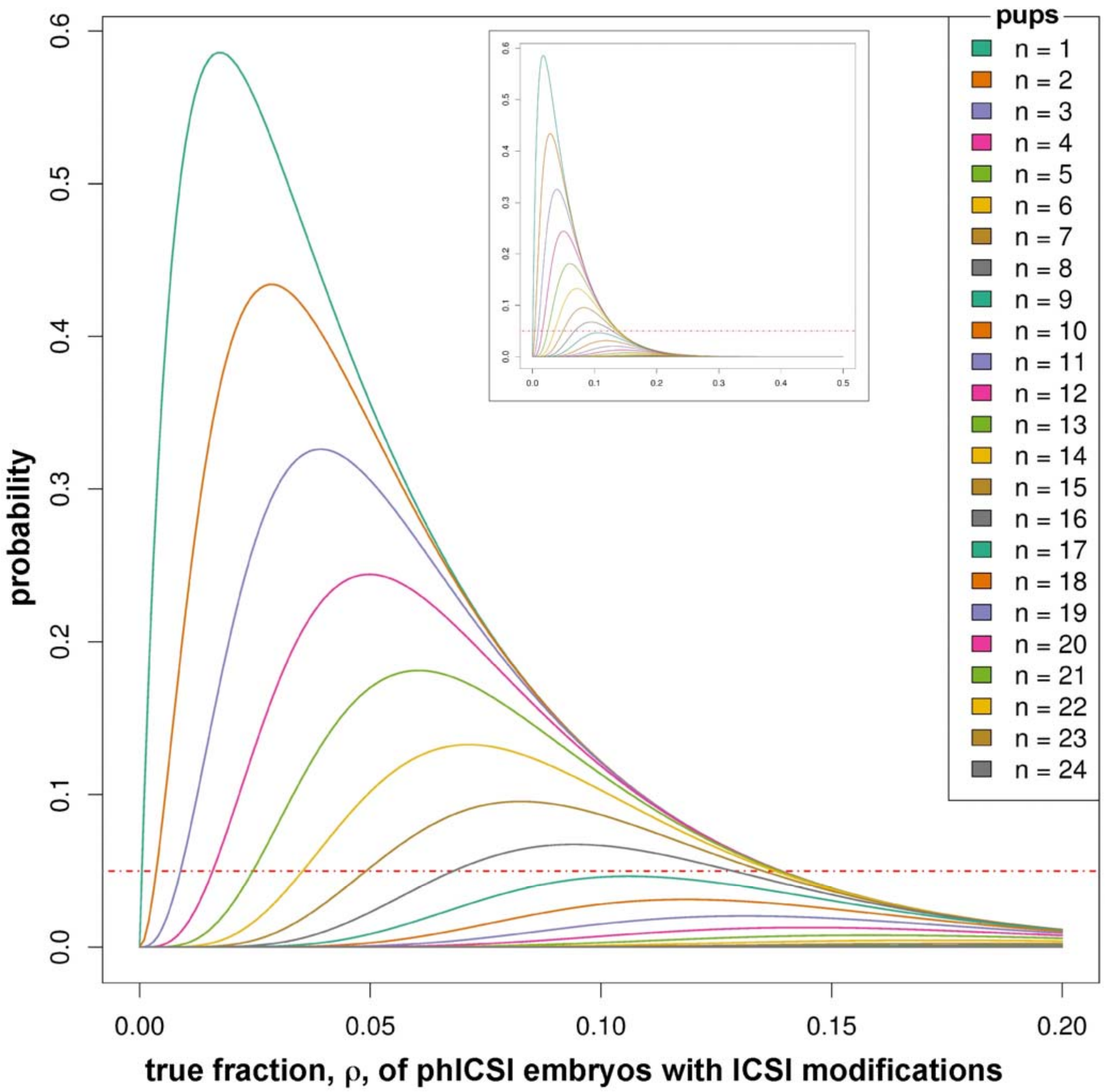

Supplementary Figure 13 Probability for ≥*n* phICSI embryos with modifications observed in ICSI. Inset shows the same plot with an extended x-axis. Probability P(n, k | ρ) for the event that at least n = 1, 2, ..., 24 embryos with ICSI modifications develop to term as a function of the unknown true fraction ρ of phICSI embryos with ICSI modifications, given the additional evidence that k = 20 out of 20 independent experiments detected phICSI modifications in an analogous population (same ρ). The production of 9 or more

phICSI embryos with modification characteristic of ICSI out of a total of 24 that developed to term can be excluded on the 5%-significance level, since P(9, 20 | ρ) ≤ 0.0465 (worst case) for all ρ. The probability that not a single embryo is phICSI modification-derived is only P(24, 20 | ρ) ≤ 9.84e-17. Details are presented in the Methods section.

Supplementary Table 1 Development of embryos *in vitro* and *in vivo*

		***in vitro* (%)**					***in vivo***					
**Treatment**	**No.**	**24 h (2C)**	**48 h (>3C)**	**72 h (mor)**	**96 h (mb)**	**120 h (blast)**	**Emb transf**	**Recips (preg)**	**Pups**	**Pups/emb transf (%)**	**At weaning Male**	**Female**
1nP	210	99.5±1.3	98.0±2.3	88.9±7.0	86.1±6.6	76.8±16.2	-	-	-	-	-	-
1nP (mock)	85	100.0±0.0	98.7±2.3	89.6±2.7	74.9±6.2	73.1±5.6	-	-	-	-	-	-
ICSI	84	97.6±3.5	97.6±3.5	97.6±3.5	97.6±3.5	94.0±1.8	106	30 (28)	85	82.5±9.5	16	14
phICSI-7	104	99.1±1.5	99.1±1.5	90.2±2.7	62.1±20.4	34.1±7.9	465	31 (4)	5	1.0±1.7	4	1
phICSI-10	105	100.0±0.0	99.0±1.7	90.7±3.4	59.2±3.9	35.5±4.4	384	21 (5)	7	1.8±1.4	2	4
phICSI-13	113	100.0±0.0	88.8±5.4	70.5±4.1	56.4±6.3	37.2±4.0	259	15 (9)	21	8.1±1.8	9	8
phICSI-*PiV*	nd	nd	nd	nd	nd	nd	154	12 (1)	2	1.2±1.2	2	0
ICSI-1bla	198	100.0±0.0	99.5±0.8	94.4±3.0	93.9±3.7	90.7±3.3	237	23 (18)	107	43.3±15.1	34	34
phICSI-13-1bla	149	100.0±0.0	85.3±13.6	61.6±6.7	43.6±10.1	21.5±6.5	232	14 (10)	24	10.4±3.1	14	10
phICSI-13-1bla-CBA	nd	nd	nd	nd	nd	nd	195	12 (8)	20	10.2±0.9	7	10
ROSI	nd	nd	nd	nd	nd	nd	50	5 (5)	16	31.7±1.6	nd	nd
phROSI	nd	nd	nd	nd	nd	nd	213	5 (12)	6	2.6±1.0	nd	nd
pnT-G1 (mtdT)	38	100.0±0.0	98.1±1.9	98.1±1.9	98.1±1.9	79.8±4.8	56	10 (5)	2	3.6±0.1	0	2
pnT-G2 (mtdT)	46	100.0±0.0	93.2±6.8	86.6±9.3	75.8±7.6	58.5±4.0	56	7 (0)	0	0	0	0
cyT-G1-1bla	nd	nd	nd	nd	nd	nd	36	4 (3)	11	31.9±11.9	nd	nd
cyT-G2-1bla	nd	nd	nd	nd	nd	nd	36	4 (4)	16	43.1±11.9	nd	nd
pnTz-G1-1bla	nd	nd	nd	nd	nd	nd	35	4 (4)	14	40.3±3.5	nd	nd
pnTz-G1-1bla	nd	nd	nd	nd	nd	nd	39	5 (4)	18	46.3±6.3	nd	nd

*Experiments were initiated on ≥2 experimental days for each treatment. 1nP, haploid parthenogenote; mock, mock-injected; nd, not determined; mor, morula; mb, morula/blastocyst; blast, blastocyst; emb tf, embryo transfer at the 2-cell stage or temporal equivalent; recips (preg), number of recipients (recipients falling pregnant). Some pups were rejected by foster mothers prior to weaning. For pnT-G1 and -G2, *mtdT* (encoding membrane tdTomato) transgenic sperm were used for *in vitro* developmental series and wt B6D2F1 sperm for *in vivo* development. All data are from ≥2 experimental days.

Supplementary Table 2 PCR primers used in this work

sequence	forward (5'→3')	reverse (5'→3')
Cre	CATTTGGGCCAGCTAAACAT	ATTCTCCCACCGTCAGTACG
mTmG-S	GCCCTCGACACTAGTGAACC	-
mTmG-mT-AS	TTCCACGATGGTGTAGTCCTC	-
mTmG-mG-AS	GAACTCCAGCAGGACCATGT	-
D1Mit200	GCCATGTTCATGTACATAGGTAGG	ATGGATGGATGGTTTTCCTG
D4Mit343	GTGGGATTTAGACTCAAGTTGACC	TATGCTAGGTGTGGAACACACA
D7Mit270	CCCTCCATCATCCTCCTTC	TCTCAAAAAGTCAATGGTGCC
D9Mit328	CATTTACTGTCTCTCTTTCATTCTCTG	CTTACATCTGGTCCACAAGAAGG
Sry	AAGCTTTGCTGGTTTTTGGA	GCAGGTGGAAAAGCCTTACA
Xist	ACCCAGTTTTCTGTGCTGCT	TTGACGATCCCTAGGTGGAG
Oct4	CCGACAACAATGAGAACCTTCAGG	ATCTGCTGTAGGGAGGGCTTCG
Nanog	GCAAGCGGTGGCAGAAAAAC	GCAATGGATGCTGGGATACTCC
Esrrb	GACATTGCCTCTGGCTACCACT	ACTTGCGCCTCCGTTTGGTGAT
GATA4	TGTGCCAACTGCCAGACTAC	TGGGCTTCCGTTTTCTGGTT
Cdx2	AGACAAATACCGGGTGGTGTA	CCAGCTCACTTTTCCTCCTGA
H3f3a	CCATGCCAAACGTGTAACAA	TACCTTTGACCCCATGGAAA
Sox2	GGAAAAAAACCACCAATCCCATCC	TTTGCGAACTCCCTGCGAAG
Wdr5	ACAGGCGGGAAGTGGATTGT	ACGCTGCTGAGGCAATGATG
Venus	ACGTAAACGGCCACAAGTTC	GAACTCCAGCAGGACCATGT
